# Genetics of environmental sensitivity and its association with variations in emotional problems, autistic traits, and wellbeing

**DOI:** 10.1038/s41380-024-02508-6

**Published:** 2024-03-18

**Authors:** Elham Assary, Olakunle A. Oginni, Genevieve Morneau-Vaillancourt, Georgina Krebs, Alicia J. Peel, Elisavet Palaiologou, Celestine Lockhart, Angelica Ronald, Thalia C. Eley

**Affiliations:** 1 Social, Genetic and Developmental Psychiatry Centre, Institute of Psychiatry, Psychology and Neuroscience, King’s College London, London, UK; 2 Department of Mental Health, Obafemi Awolowo University, Ile-Ife, Nigeria; 3 Division of Psychological Medicine and Clinical Neurosciences, Wolfson Centre for Young People’s Mental Health, Cardiff University, Cardiff, UK; 4 Research Department of Clinical, Educational and Health Psychology, University College London, London, UK; 5 National and Specialist OCD, BDD and Related Disorders Clinic for Young People, South London and Maudsley NHS Foundation Trust, London, UK; 6 School of Psychology, University of Surrey, Guildford, UK; 7 UK National Institute for Health Research (NIHR) Biomedical Research Centre, South London and Maudsley Hospital, London, UK

**Keywords:** Psychology, Genetics

## Abstract

Greater environmental sensitivity has been associated with increased risk of mental health problems, especially in response to stressors, and lower levels of subjective wellbeing. Conversely, sensitivity also correlates with *lower* risk of emotional problems in the *absence* of adversity, and in response to *positive* environmental influences. Additionally, sensitivity has been found to correlate positively with autistic traits. Individual differences in environmental sensitivity are partly heritable, but it is unknown to what extent the aetiological factors underlying sensitivity overlap with those on emotional problems (anxiety and depressive symptoms), autistic traits and wellbeing. The current study used multivariate twin models and data on sensitivity, emotional problems, autistic traits, and several indices of psychological and subjective wellbeing, from over 2800 adolescent twins in England and Wales. We found that greater overall sensitivity correlated with greater emotional problems, autistic traits, and lower subjective wellbeing. A similar pattern of correlations was found for the Excitation and Sensory factors of sensitivity, but, in contrast, the Aesthetic factor was positively correlated with psychological wellbeing, though not with emotional problems nor autistic traits. The observed correlations were largely due to overlapping genetic influences. Importantly, genetic influences underlying sensitivity explained between 2 and 12% of the variations in emotional problems, autistic traits, and subjective wellbeing, *independent* of trait-specific or overlapping genetic influences. These findings encourage incorporating the genetics of environmental sensitivity in future genomic studies aiming to delineate the heterogeneity in emotional problems, autistic traits, and wellbeing.

## Introduction

Some individuals are generally more sensitive to the quality of their environment than others, and it has been suggested that sensitivity operates in a ‘for better and for worse’ manner [1, 2]. From this perspective, greater sensitivity could increase the potential for adverse outcomes in response to stressors, but also for favourable outcomes in response to positive influences such as psychological interventions [3]. Environmental sensitivity has been conceptualised as a personality trait [4] and is under moderate genetic influence [5]. Although high sensitivity is not considered to be a disorder [6], growing evidence suggests that it is associated with traits relating to both mental health conditions and neurodiversity.

In line with differential susceptibility to the environment model [2], rather than mere vulnerability to stressors, greater sensitivity has also been found to be associated with lower risk of mental health problems in the absence of adversity [7, 8]. The associations with both positive and negative outcomes paint a complex picture of sensitivity. Indeed, it is difficult to discern, due to paucity of research, what drives the positive associations with sensitivity and how it may relate to different aspects of wellbeing. Importantly, it remains unknown the extent to which the associations between sensitivity, emotional problems, autistic traits and wellbeing reflect a shared aetiology, and whether the genetic influences on environmental sensitivity explain individual differences in these outcomes. Thus, the aim of the current study was to examine the aetiological overlap between environmental sensitivity and these traits, and also to estimate the extent to which genetics of environmental sensitivity influence variations in these outcomes.

In recent years, different instruments have been developed to quantify environmental sensitivity as a personality trait, including questionnaires and observational measures. There are differences among these instruments such as number of items [9], informant source [6, 10], and its underlying factor structure [11]. But, they broadly conceptualise environmental sensitivity as a stable personality type (highly sensitive personality), marked by generally greater reactivity to emotional, psychological, and sensory stimuli, greater attention to aesthetics and details, and overstimulation [6, 12]. Using this conceptualisation of sensitivity, research has found greater sensitivity is associated with generally poorer mental health outcomes. For example, in adults and adolescents, this includes affective difficulties such as anxiety and depressive symptoms and emotional regulation problems [13–16]. Highly sensitive adults also report poorer physical health [17, 18], higher risk of burn-out syndrome [19], and require longer psychological recovery time in response to stressors [20]. Recent studies also show that higher sensitivity is associated with emotional problems and higher reactive stress e.g., in response to the COVID-19 pandemic [21, 22].

Little is known about the mechanisms that underlie the associations with mental health problems, though research with children suggests that sensitivity may act as a *moderator* to intensify the impact of environmental exposures on a range of outcomes. For example, highly sensitive children are more affected by their home and school environments, in both positive and negative directions. Specifically, sensitivity has been associated with increased externalising problems in the context of negative parenting practices but decreased externalising problems in the presence of positive parenting [10, 23]. Similarly, highly sensitive children who experience childhood adversity report lower physical comfort and lower self-efficacy, whereas those growing up in supportive home environments exhibit greater than average self-efficacy [24]. With regards to school environment, a study of over 2000 children found that there was a greater reduction in victimisation and emotional symptoms following a bullying intervention for boys who scored higher on the sensitivity measure, compared to those who scored lower [25]. Genetic studies have also implicated sensitivity as a potential moderator of risk for mental health problems in response to environmental risk. For example, a polygenic score of sensitivity was found to moderate responses to parenting practices. Specifically, for children with a high genetic sensitivity score, negative parenting was associated with higher emotional problems, and positive parenting with lower emotional problems [26].

Interestingly, sensitivity has also been associated with neurodevelopmental disorders, albeit less frequently than emotional problems. For example, higher scores on the highly sensitive personality questionnaires are correlated with greater autistic-like traits [14, 27, 28]. This association may partly reflect the fact that both traits include sensory sensitivity symptoms that may be measured in both highly sensitive personality and autism screening questionnaires. However, greater sensitivity has been associated with socio-communication difficulties aspect of autistic traits, which do not include the sensory sensitivity items [14]. Therefore, the association between environmental sensitivity and autistic traits are not solely due to overlapping items assessing sensory sensitivity in both instruments. While the source of the observed association is undetermined, it is plausible that a genetic predisposition to be generally more sensitive to one’s physical and psychosocial environment also overlaps with risk for other difficulties, such as social communication issues, a hallmark of autism.

We note that sensitivity has also been related to *lower risk* of mental health problems and *enhanced functioning* in the absence of adversity and in nurturing contexts. Though still preliminary, there is some indication that this may be due to sensitivity being associated with other characteristics that reflect psychological wellbeing. For example, highly sensitive individuals tend to report having higher levels of mindfulness, empathy, introspection and meaning in life [29]. Research into the factor structure of sensitivity offers further insights. Specifically, factor analysis of the highly sensitive personality measures has typically found three factors; “Excitation”, “Sensory” and “Aesthetic” sensitivities [4, 30, 31] (though some have found different number of factors or only one, see for example [6, 11]). The *Excitation* factor relates to becoming easily overwhelmed by external stimuli and the *Sensory* factor reflects unpleasant arousal to stimuli such as noises and textures whereas the *Aesthetic* factor reflects greater attention to aesthetics and deriving more pleasure from positive stimulations. While the excitation and sensory factors correlate with risk for more negative outcomes such depressive and anxiety symptoms, poor social communication skills [14], and negative affect and lower subjective wellbeing [32], the Aesthetic factor is associated with traits that are generally considered positive. These include characteristics such as openness to experiences, agreeableness, conscientiousness, and positive affect [32], and better communication skills [14]. Greater sensitivity may therefore relate to more positive outcomes via enhancements in paying attention to and taking more pleasure from contexts and exposures that are nurturing. In support of this, a recent study found that genetic factors underlying sensitivity partly overlap with those influencing both positive and negative appraisals of life events [33]. It is therefore possible that the genetic influences on sensitivity and subjective experiences of life events contribute to individual differences in risk for developing mental health problems or recovering from them.

Sensitivity is heritable [5] and has been shown to be correlated with mental health outcomes such as emotional problems (anxiety, and depressive symptoms), autistic traits, and wellbeing. Despite this, there are no studies to date that have examined their aetiological overlap. Examining the source of these correlations is important, because evidence of a shared genetic aetiology further strengthens the case to interrogate molecular genetics of this trait and the underlying mechanisms that explain heterogeneity in risk for mental health problems as a function of variations in sensitivity to environmental contexts. Also importantly, studying the associations between sensitivity and a broad range of wellbeing measures may shed some light on the least understood aspect of sensitivity that relates to more positive outcomes. Examining these questions in an adolescent sample is particularly valuable for emotional problems that typically have an adolescence age of onset.

The main aims for the current study, as pre-registered here, were to (a) examine the extent of genetic and environmental contributions to the phenotypic correlations between sensitivity and emotional problems (anxiety and depressive symptoms), autistic traits, and wellbeing; and (b) estimate the extent to which the aetiological influences on sensitivity also explain variations in these outcomes, independent of those that are specific to each trait or shared between them. We hypothesised that both genetic and environmental factors would contribute to the correlations between sensitivity and emotional problems, autistic traits, and wellbeing; and that aetiological factors associated with sensitivity would partly explain the variation in these outcomes.

## Methods

### Sample

Participants came from the Twins Early Development Study (TEDS), a sample of over 15,000 twin pairs born in England and Wales between 1994 and 1996 [34]. The twins have been followed longitudinally throughout childhood into adulthood. Cognitive, emotional, and behavioural data have been collected from the twins, their parents, and teachers, at regular time points. The sample for the current study included a subset of these families who participated in different waves of data collection when twins were between the ages of 15–17 years (see supplementary information for more details). In accordance with standard exclusion criteria for TEDS analyses, participants with severe medical disorders, severe perinatal complications, unknown demographic variables or zygosity were excluded. The final sample included data on 2944 twins (518 MZ and 1039 DZ twin pairs; 1702 females), with data on environmental sensitivity. Of those with environmental sensitivity data, 2944 also had data on anxiety symptoms, 2941 on depressive symptoms and 2940 on autistic traits. Data on wellbeing were only available for a subset of these individuals (*N* = 1163). Mean age at the time of data collection for sensitivity measure was 17.06 years old (sd = 0.88 years). Informed consent was obtained from all participants.

### Measures

*Environmental sensitivity* was measured with the Highly Sensitive Child scale [4], a 12-item self-report questionnaire devised specifically to measure sensitivity in children and adolescents. The scale measures participants’ endorsement of statements such as “When someone observes me, I get nervous. This makes me perform worse than normal”. Factor analysis of the measure has identified three factors: “Excitation” factor includes items that relate to becoming mentally overwhelmed by external stimuli (e.g., “I am annoyed when people try to get me to do too many things at once”). The *“Sensory”* factor includes items that relate to unpleasant sensory arousal to external stimuli (e.g., “Loud noises make me feel uncomfortable”) and the “*Aesthetic”* factor includes items that relate to aesthetic awareness such as “I notice it when small things have changed in my environment”. The responses are on a Likert rating scale ranging from 1 = not at all to 7 = extremely. The Cronbach’s alpha in the current sample was 0.81.

*Anxiety symptoms* were measured with 19 items from the Anxiety Related Behaviours Questionnaire ARBQ [35]; a parent-report questionnaire that asks parents to rate statements such as my child “is anxious that bad things will happen”, and “often seems worked up, on edge or tense”, on a scale of 1=not true to 3= very true. The Cronbach’s alpha in the current sample was 0.86.

*Depressive symptoms* were measured with 13 items from the short Mood and Feelings questionnaires [36], a self-report measure assessing participants’ mood and feelings. Example items are “I felt miserable or unhappy” and “I hated myself” rated on a scale of 1=not true, to 3=very true. The Cronbach’s alpha in the current sample was 0.91.

*Autistic traits* were measured by the abbreviated Autism Spectrum Quotient (AQ) questionnaire [37], which consisted of 13 self-report items that index autistic characteristics as reflected in difficulties with social communication, and greater attention to detail. Example items are “I prefer to do things with others rather than on my own” and “I usually notice car number plates or similar strings of information” rated on a scale of 0=definitely disagree, to 3=definitely agree. Items were reverse coded as necessary to ensure the total score reflected higher levels of autistic traits. The Cronbach’s alpha in the current sample was 0.79.

*Wellbeing* was measured via eight self-report questionnaires that relate to different aspects of wellbeing such as subjective/hedonic and psychological/eudaimonic wellbeing. The wellbeing indices included ambition, curiosity, grit, optimism, gratitude, happiness, life satisfaction and hopefulness (see supplementary information for descriptions of each measure, and Table S1a and S1b for descriptive statistics and correlations). To avoid running the analyses with multiple correlated outcomes, and to consolidate the data captured by these various indices of wellbeing, a principal component analysis was conducted on all eight measures. Two principal components emerged (Fig. S1), with one component reflecting subjective wellbeing (indicated by high factor loadings from life satisfaction, happiness, optimism, gratitude), and another component reflecting psychological wellbeing (indicated by high factor loadings from grit, ambition, curiosity, hopefulness). Using the regression method, individuals’ scores on the two principal components were extracted to indicate subjective wellbeing and psychological wellbeing factor scores in the planned analyses.

### Data analysis

Study protocol was approved by TEDS steering committee, and the analyses were pre-registered here. First, descriptive statistics, correlations between all study variables and distribution of data were examined (see supplementary information Table S2a and b). All variables were regressed for age and sex prior to running the multivariate ACE analyses, as is customary for twin analyses [38]. Variables (i.e., depressive symptoms, and parent and self -report anxiety symptoms) with skew >1 were transformed, using rank transformation, to meet normality assumptions. Only correlations that were greater than *r* = 0.2 were taken forward into the twin analyses.

Second, two variants of the multivariate ACE model, the Cholesky decomposition, were constructed to test the main aims of the current paper. The standard twin ACE model partitions the phenotypic variance into additive genetic effects (A), shared/common environmental effects (C) and non-shared environmental effects (E). Shared environmental effects are the environmental influences that contribute to the similarity between twins, whereas non-shared environments are the environmental influences that make twins dissimilar, such as individual-specific life events. The genetic correlation (r_A_) between MZ and DZ twin pairs is assumed to be 1 and 0.5, respectively. The correlation between twins’ shared environments (r_C_) is assumed to be 1 for both MZ and DZ twin pairs. Higher phenotypic similarity within MZ twin pairs, in comparison with DZ twin pairs, can therefore be attributed to MZ twins’ higher genetic similarity (A). Shared environmental influences (C) are indicated when the phenotypic correlations between MZ twin pairs is comparable to that between DZ twin pairs. However, if the MZ correlation is higher than twice the DZ correlation, non-additive genetic effects, such as dominance (D) are indicated. Non-shared environmental influences (E) are estimated as the difference between the MZ twin correlations and 1. Non-shared environmental influences (E) also include measurement error.

Multivariate models are an extension of univariate ACE models, whereby the cross-twin cross-trait correlations are examined to infer the role of genetic and environmental influences on the associations between traits. It is possible to parse the variance/covariance of the phenotypes of interest into two sets of ACE effects: those that are due to overlapping ACE influences and those that are due to distinct ACE influences for each phenotype.

To examine aim 1, a correlated factors solution of a multivariate Cholesky decomposition model was specified (Model 1: correlated factors solution of the Cholesky decomposition model). This is a variation of the Cholesky model whereby the genetic and environmental paths between variables are interpreted as genetic and environmental correlations.

To test aim 2, a different variation of the multivariate Cholesky model was specified (Model 2: ordered- correlated factors solution of the Cholesky decomposition model). In this model, the associations between sensitivity and each of the other measures (anxiety, and depressive symptoms, autistic traits, and wellbeing) were interpreted as Cholesky decomposition paths, whereas the associations among these four were interpreted as a correlated factor solution. This model indicates how much of the variance in anxiety symptoms, depressive symptoms, autistic traits and wellbeing is accounted for by genetic and environmental influences on sensitivity, and how much of their variance is attributable to genetic and environmental influences not shared with sensitivity (Fig. 2).

According to power calculations for twin models [39], the current sample would be sufficiently (>80%) powered to detect medium to large genetic correlations in multivariate models.

Two sensitivity analyses were run. In the first, the parent-report measure of anxiety in models 1 and 2 was replaced with a self-report measure, the Childhood Anxiety Sensitivity Index CASI; [40]. This measure mainly captures adolescents’ awareness of, and tendency to negatively interpret, symptoms of anxiety [41]. Second, the total environmental sensitivity score was replaced in turn with each of its three factors (“Excitation”, “Aesthetic”, “Sensory”) in models 1 and 2, to examine their association with mental health outcomes and wellbeing. In line with previous research, we expected Aesthetic factor to be more strongly/positively associated with wellbeing, and the other two with more emotional problems and autistic traits.

We also conducted a post-hoc analysis that examined whether sensitivity was associated with differences in variance for these traits, since sensitivity is expected to play a role in their variability. This was done by examining the variance of depression, anxiety, autistic traits, and subjective wellbeing measures (happiness, life satisfaction, optimism, hopefulness) in the low versus high sensitivity groups (25th vs 75th percentiles of the scores, respectively).

Genetic model fittings were conducted within R [42], using the OpenMx [43] package.

## Results

### Descriptive statistics

The descriptive statistics for the study variables are presented in Table S2a. The correlations between study variables are presented in Table 1. As expected, overall sensitivity was moderately (*r* ~0.32, *p* < 0.001) and positively correlated with anxiety symptoms, depressive symptoms, and autistic traits. Sensitivity was also associated moderately, but negatively, with the subjective wellbeing factor (*r* = −0.32, *p* < 0.001), and to a lesser extent with the psychological wellbeing factor (*r* = −0.09, *p* < 0.001). Only the subjective wellbeing factor was included in the follow up twin analyses, as the phenotypic correlation between sensitivity and psychological wellbeing was less than *r* = 0.2 threshold.

**Table 1:** Correlation between study variables.

	Sensitivity	Aesthetic	Excitation	Sensory	Anx	Dep	AUT	Subj well
Aesthetic	0.58*							
Excitation	0.89*	0.28*						
Sensory	0.74*	0.18*	0.54*					
Anxiety symptoms	0.21*	−0.02	0.25*	0.19*				
Depressive symptoms	0.35*	0.07	0.38*	0.25*	0.29*			
Autistic traits	0.32*	0.04	0.34*	0.30*	0.31*	0.32*		
Subjective wellbeing	−0.32*	0.09*	−0.42*	−0.24*	−0.31*	−0.65*	−0.44*	
Psychological wellbeing	−0.09*	0.24*	−0.24*	−0.06*	−0.18*	−0.17*	−0.09*	0.43*

*Sensitivity* sensitivity total score, *Aesthetic* Aesthetic factor of sensitivity, *Excitation* Excitation factor of sensitivity, *Sensory* Sensory factor of sensitivity, *AUT* autistic traits, *Dep* depressive symptoms, *Anx* anxiety symptoms, *Subj well* subjective wellbeing factor score.

*p < 0.001.

The results of sensitivity analyses that examined the correlations between the three factors of sensitivity (Excitation, Sensory, Aesthetic) and other study variables are presented in Table S2b. Excitation and Sensory factors of sensitivity showed similar patterns of associations as the total sensitivity score (e.g., moderate positive associations with emotional problems and autistic traits, and negative with subjective wellbeing). However, correlations for the Aesthetic factor were different (e.g., non-significant/small and negative with emotional problems, autistic traits, but positive with psychological and subjective wellbeing). This pattern of correlations was as expected given the Aesthetic factor is thought to index the positive side of sensitivity. Therefore, the follow up twin analyses with the Aesthetic factor was a bivariate model with psychological wellbeing as the only variable with correlation >0.2.

Also, as expected, we found significant differences in variance of depression and anxiety symptoms, autistic traits, and subjective wellbeing measures (expect for optimism), depending on sensitivity. Specifically, high sensitivity was associated with greater variance in these outcomes than low sensitivity (see supplementary information Table S3a–c).

### Genetic overlap between sensitivity, emotional problems, autistic traits, and subjective wellbeing

**Fig. 1:**
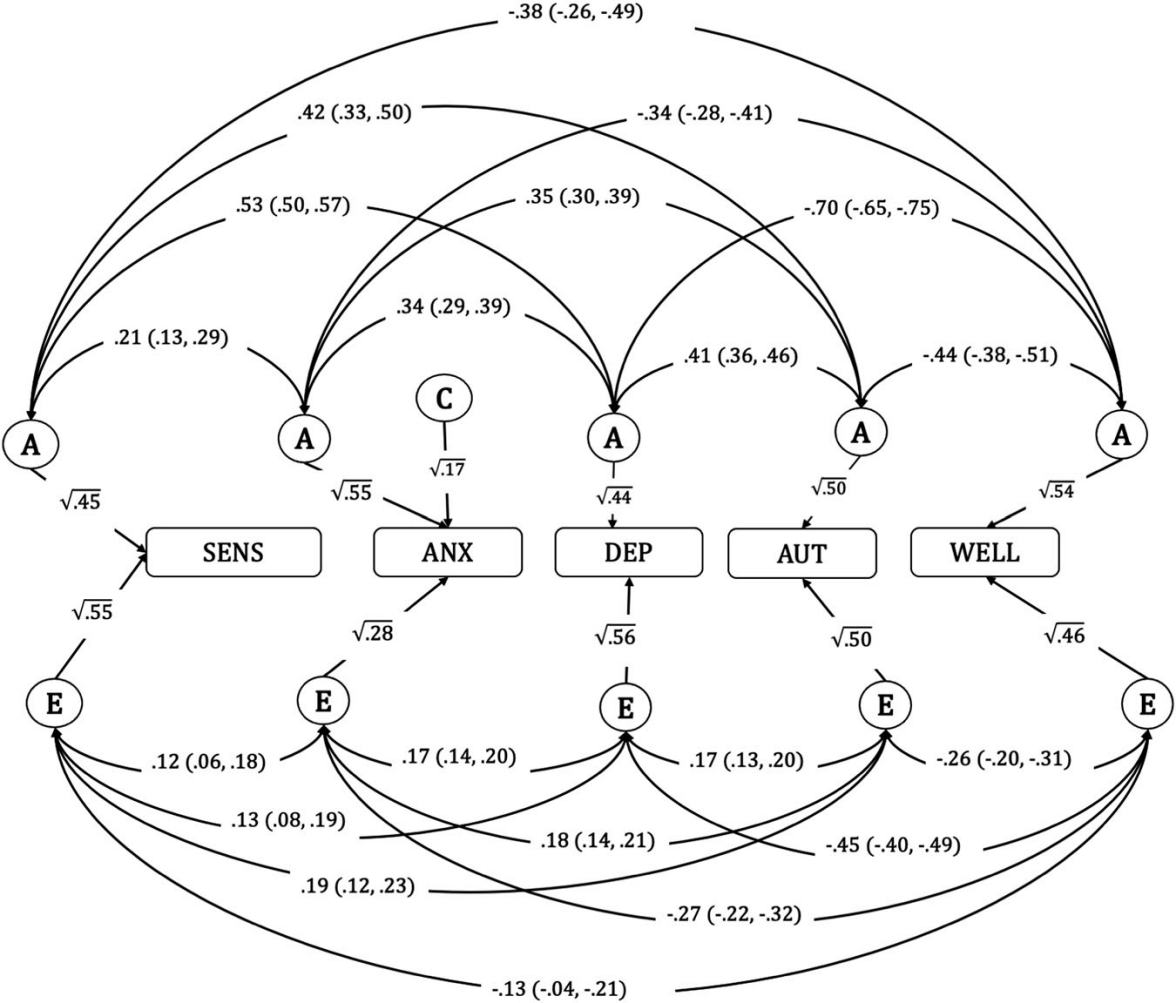
Model 1 (Cholesky decomposition correlated factors solution model) showing genetic and environmental correlations between sensitivity, anxiety, and depressive symptoms, autistic traits, and subjective wellbeing. SENS environmental sensitivity, ANX anxiety symptoms, DEP depressive symptoms, AUT autistic traits, WELL subjective wellbeing factor, A additive genetic influences, C common environmental influences, E unique environmental influences. 95% Confidence intervals are shown in parentheses. Straight arrows represent contributions from ACE components to each variable, and curved arrows represent correlations between ACE components for each variable.

Greater cross-twin, cross-trait correlations among MZ than DZ twins suggested genetic influences operate on the relationship between sensitivity, emotional problems, autistic traits, and wellbeing (see Table 2). The results of multivariate ACE model 1 (correlated factors solution of the Cholesky decomposition model) are presented in Fig. 1. There were no evidence of common environmental influences (C) contributing to any of the variables, with C paths estimated as 0, except for anxiety (c^2^ = 0.17). Therefore, all C paths were dropped, except for a C path for anxiety to ensure best model fit (supplementary information Table S4).

**Table 2:** Cross-twin Cross-trait correlations for sensitivity, anxiety symptoms, depressive symptoms, autistic traits, and subjective wellbeing.

	Correlation with	rMZ	MZ Cross-trait	rDZ	DZ Cross-trait
Sensitivity		0.46 (0.43, 0.51)		0.21 (0.16, 0.24)	
	Anx		0.10 (0.08, 0.12)		0.07 (0.05, 0.09)
	Dep		0.22 (0.19, 0.26)		0.15 (0.13, 0.17)
	AUT		0.19 (0.17, 0.22)		0.12 (0.10, 0.14)
	Well		−0.18 (−0.13, −0.21)		−0.12 (−0.09, −0.15)
Anxiety symptoms		0.72 (0.71, 0.73)		0.45 (0.44, 0.48)	
	Dep		0.16 (0.14, 0.18)		0.10 (0.09, 0.12)
	AUT		0.18 (0.17, 0.20)		0.10 (0.08, 0.12)
	Well		−0.18 (−0.21, −0.16)		−0.12 (−0.09, −0.15)
Depressive symptoms		0.42 (0.40, 0.44)		0.27 (0.25, 0.30)	
	AUT		0.19 (0.17, 0.21)		0.12 (0.10, 0.13)
	Well		−0.32 (−0.34, −0.30)		−0.22 (−0.19, −0.24)
Autistic traits		0.53 (0.51, 0.55)		0.21 (0.19, 0.22)	
	Well		−0.24 (−0.26, −0.20)		−0.10 (−0.09, −0.13)
Subjective wellbeing		0.53 (0.51, 0.57)		0.30 (0.27, 0.34)	

*Anx* anxiety symptoms, *AUT* autistic traits, *Dep* depressive symptoms, *Well* subjective wellbeing, *rMZ* monozygotic (MZ) twin correlations, *rDZ* dizygotic (DZ) twin correlation, numbers in parentheses represent 95% confidence intervals.

The path estimates from the best fitting AE model, as shown in Fig. 1, indicate the extent to which the genetic and environmental influences on each trait are correlated. There were positive genetic correlations between sensitivity and anxiety symptoms (*r*_g_ = 0.21), depressive symptoms (*r*_g=_ 0.53), autistic traits (*r*_g=_ 0.42), and negative genetic correlations with subjective wellbeing (*r*_g_ = −0.38). There was also evidence of correlating non-shared environmental influences between sensitivity and these outcomes, though to a lesser extent (*r*_A_ = 0.12,0.13, 0.19 and −0.13, respectively).

Table 3 shows the extent to which the phenotypic correlations (*r*_Ph_) between sensitivity, anxiety symptoms, depressive symptoms, autistic traits, and subjective wellbeing are due to these correlating genetic (*r*_PhA_) and environmental influences (*r*_PhE_). For example, of the total phenotypic correlation (*r*_Ph_ = 0.15) between sensitivity and anxiety, two-thirds (0.10) of the correlation were due to overlapping genetic factors, and one-third (0.05) was due to overlapping environmental factors. We found similar results for the self-report measure of anxiety, whereby 68% of the phenotypic correlation between sensitivity and anxiety (*r*_Ph_ = 0.41) were due to overlapping genetic effects (see Table S5b and Fig. S2).

**Table 3:** Model 1 (correlated factors solution of the Cholesky decomposition model) results.

	*r*_Ph_	*r*_PhA_	*r*_PhE_	% *r*_PhA_	% *r*_PhE_
**Sensitivity**					
Anxiety symptoms	0.15 (0.13, 0.17)	0.10 (0.07, 0.14)	0.05 (0.02, 0.07)	67	33
Depressive symptoms	0.31 (0.30, 0.34)	0.24 (0.20, 0.27)	0.07 (0.04, 0.11)	77	23
Autistic traits	0.30 (0.28, 0.32)	0.20 (0.20, 0.23)	0.10 (0.06, 0.12)	67	33
Subjective wellbeing	−0.25 (−0.22, −0.28)	−0.19 (−0.14, −0.24)	−0.06 (−0.02, −0.11)	76	24
**Anxiety symptoms**					
Depressive symptoms	0.23 (0.22, 0.24)	0.16 (0.14, 0.19)	0.07 (0.06, 0.08)	70	30
Autistic traits	0.25 (0.24, 0.26)	0.18 (0.16, 0.20)	0.07 (0.05, 0.08)	72	28
Subjective wellbeing	−0.29 (−0.27, −0.31)	−0.19 (−0.15, −0.22)	−0.10 (−0.08, −0.12)	66	34
**Depressive symptoms**					
Autistic traits	0.28 (0.27, 0.29)	0.19 (0.17, 0.22)	0.09 (0.07, 0.11)	68	32
Subjective wellbeing	−0.57 (−0.56, −0.58)	−0.34 (−0.31, −0.37)	−0.23 (−0.20, −0.25)	60	40
**Autistic traits**					
Subjective wellbeing	−0.35 (−0.34, −0.37)	−0.23 (−0.20, −0.27)	−0.12 (−0.10, −0.15)	66	34

Results from the best fitting AE model; *r*_Ph_= phenotypic correlation; *r*_PhA_= phenotypic correlation due to correlating genetic effects; *r*_PhE_= phenotypic correlation due to correlating environmental effects; % *r*_PhA_= percentage of phenotypic correlation due to correlating genetic effects; % *r*_PhE_= percentage of phenotypic correlation due to correlating environmental effects; numbers in parentheses represent 95% confidence intervals; Percentages are rounded up.

For depressive symptoms, overlapping genetic factors accounted for 77% of the of the phenotypic correlations with sensitivity (*r*_*Ph*_ = 0.31) and environmental factors accounted for 23%. For autistic traits, genetic factors accounted for 67% of the phenotypic correlation (*r*_Ph_ = 0.30), and environmental factors 33%. Similar results were found for subjective wellbeing, whereby genetic factors explained 76% of the phenotypic correlation (*r*_Ph_ = −0.25) with sensitivity, and environmental factors 24%.

The results of the sensitivity analyses, using the Excitation and Sensory factors of sensitivity, showed a similar pattern to the total score of sensitivity, with ~68% of the phenotypic correlation between Excitation, emotional problems, autistic traits, and subjective wellbeing due to overlapping genetic influences (see Table S6b, and Fig. S3). For the Sensory factor, overlapping genetic influences explained 71%, 68%, 85 and 75% of the phenotypic correlations with anxiety symptoms, autistic traits, depressive symptoms, and subjective wellbeing, respectively (see Table S7b, and Fig. S4). The results of the bivariate twin model of the Aesthetic factor and psychological wellbeing indicated that genetic and non-shared environmental influences contributed an almost equal amount to the phenotypic correlation between sensitivity and psychological wellbeing (*r*_Ph_ = 0.23, 95%CI = [0.18, 0.29]), with overlapping genetic factors explaining 57% and environmental factors explaining 43% of the phenotypic correlation (see Table S8b and Fig. S5).

### Contribution of sensitivity to variation in anxiety symptoms, depressive symptoms, autistic traits, and subjective wellbeing

**Fig. 2:**
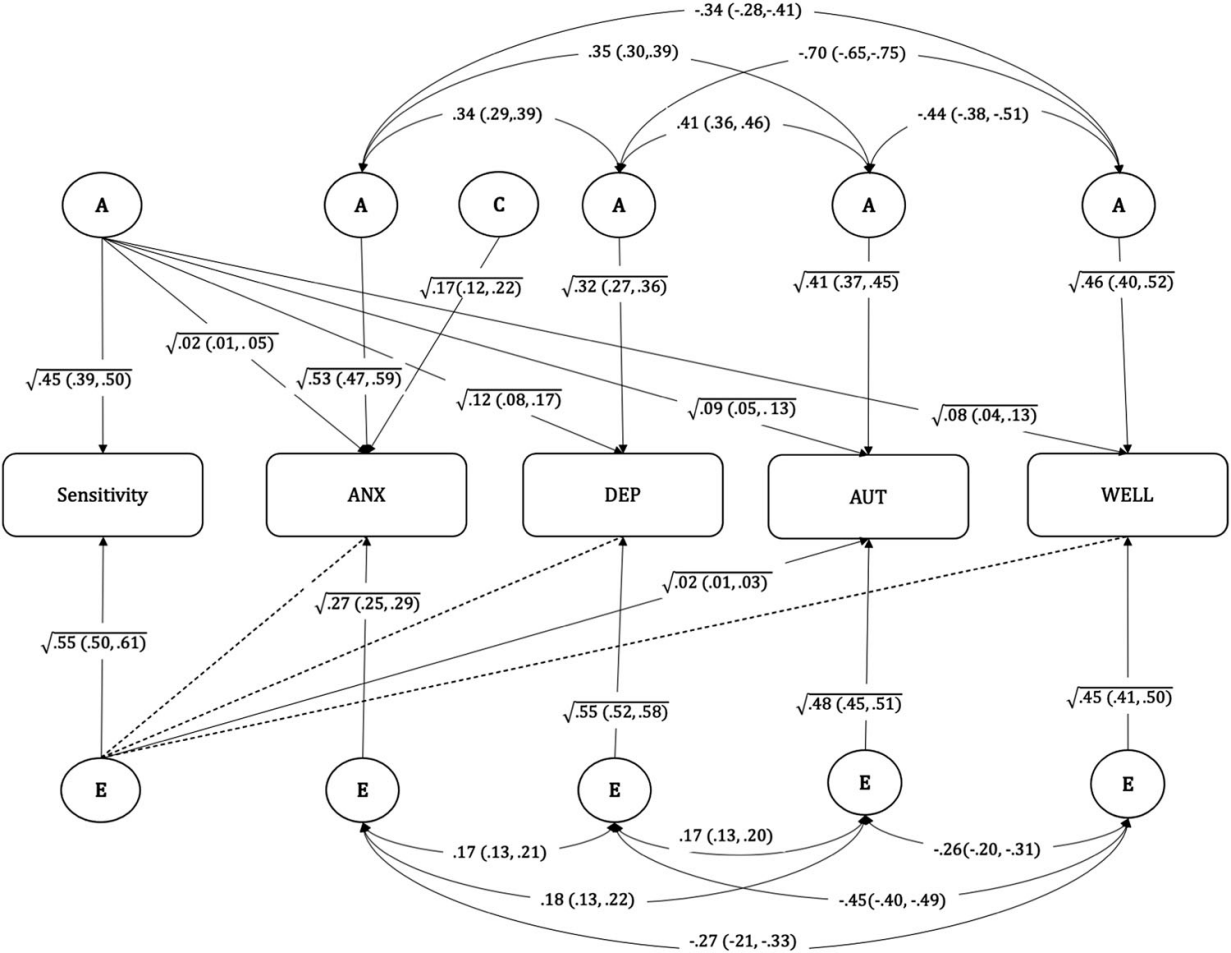
Model 2 (Cholesky decomposition ordered -correlated factors solution model) showing the contribution of genetic and environmental influences from sensitivity to anxiety, and depressive symptoms, autistic traits, and subjective wellbeing. Sensitivity environmental sensitivity, ANX anxiety symptoms, DEP depressive symptoms, AUT autistic traits, WELL subjective wellbeing score, A additive genetic influences, C common environmental influences, E unique environmental influences. Straight arrows represent contributions from ACE components to the variable, and curved arrows represent correlations between ACE components for each variable. The dotted lines indicate non-significant path estimates.

The results of the multivariate Model 2 (ordered- correlated factors solution of the Cholesky decomposition model) are presented in Fig. 2. The aim of this analysis was to estimate to what extent the genetic and environmental factors underlying sensitivity independently explain variations in emotional problems, autistic traits and subjective wellbeing.

*Anxiety symptoms:* the results indicated that genetic factors associated with sensitivity accounted for 2% of the variance of anxiety symptoms, which has a heritability of 55%. As such, genetic influences underlying sensitivity explained ~4% of the total heritability of anxiety. There were no significant contributions from environmental influences underlying sensitivity to anxiety. The analyses using the self-report anxiety measure found that genetic factors underlying sensitivity accounted for 18% of the variance of anxiety symptoms which had a heritability of 42%, thus genetic factors underlying sensitivity account for 43% of the total heritability of this measure. Environmental factors underlying sensitivity accounted for 3% of the variance of the self-report measure of anxiety symptoms (supplementary information Fig. S6).

*Depressive symptoms*: the results indicated that genetic factors associated with sensitivity accounted for 12% of the variation in depressive symptoms which has a heritability of 44%. In other words, genetic influences underlying sensitivity explained 27% of the total heritability of depressive symptoms. There were no significant contributions from unique environmental influences underlying sensitivity to depressive symptoms.

*Autistic traits:* the results indicated that genetic factors associated with sensitivity accounted for 9% of the variance in autistic traits, which is 18% of the total heritability of autistic traits at 50%. There was also a small but significant contribution (2% of variance) from environmental influences underlying sensitivity to variation in autistic traits. Together, genetic and environmental influences on sensitivity explained 11% of variation in autistic traits, independent of those distinct to autistic traits and those shared with other traits.

*Subjective wellbeing*: the results indicated that genetic influences associated with sensitivity accounted for 8% of the heritability of subjective wellbeing factor which was 54%. In other words, genetic influences underlying sensitivity explained 14% of the total heritability of subjective wellbeing. There were no significant contributions from environmental influences underlying sensitivity to subjective wellbeing.

The sensitivity analyses that replaced the total score of sensitivity with its two factors (Excitation and Sensory) found similar results as the total score, with Excitation generally showing stronger associations that the sensory factor (supplementary information Figs. S7 and S8).

## Discussion

Our study of adolescent twins identified a positive association between greater overall sensitivity and elevated risk for emotional problems and autistic traits, as well as lower levels of subjective wellbeing, but not psychological wellbeing. Examination of the three factors of sensitivity revealed a similar pattern of findings for Excitation and Sensory factors, but in contrast, the Aesthetic factor was positively associated with psychological wellbeing but not emotional problems or autistic traits. We also found variances of emotional problems, autistic traits and subjective wellbeing measures (except optimism), differed depending on sensitivity, indicating that sensitivity plays a role in variability in these traits [44], perhaps via moderating environmental exposures. Multivariate twin analyses demonstrated that shared genetic influences explained over two-thirds of the correlations between sensitivity and emotional problems, autistic traits and subjective wellbeing. The results also estimated that genetic factors related to variations in sensitivity partially accounted for individual differences in anxiety symptoms (2%), depressive symptoms (12%), autistic traits (9%), and subjective wellbeing (8%). This genetic contribution from sensitivity was over and above those that are specific to each trait or shared between them. Environmental factors involved in variation in sensitivity also explained a further 2% of individual differences in autistic traits. The correlation between aesthetic sensitivity and psychological wellbeing was due to almost equal proportion of overlapping genetic and environmental influences.

Genetic or environmental correlation may reflect genetic or environmental pleiotropy, whereby the same/correlating aetiological factors influence both phenotypes, either directly (horizontal pleiotropy), or by giving rise to an intermediate phenotype, as a consequence of which, the second phenotype develops (vertical pleiotropy). Although it is typically difficult to discern which type of pleiotropy is in play, evidence of genetic overlap is an initial step in disentangling co-morbidity and heterogeneity in risk for various phenotypes and how they may be mechanistically related. Our findings that genetics of sensitivity is relevant to heterogeneity in emotional problems is supported by previous research that found a higher genetic score of sensitivity to be associated with greater emotional problems in children in the context of negative parenting practices [26]. We interpret the findings of the genetic factors of sensitivity partially explaining variation in emotional problems, to mean that being more genetically sensitive to one’s environment may increase risk for emotional problems, perhaps by exacerbating the impact of negative environmental exposures [45]. This interpretation is supported by recent research in adolescents, showing that higher sensitivity is genetically associated with a greater tendency to evaluate the impact of environmental exposures, such as adverse life events, as stressful [33].

Our association between higher overall sensitivity and lower subjective wellbeing is in keeping with previous research on life satisfaction and sensitivity [46], but we also found that aesthetic sensitivity is associated positively with psychological wellbeing. These findings paint a more complex picture of sensitivity and wellbeing, whereby different aspects of sensitivity are associated with specific facets of wellbeing. Specifically, sensitive individuals are more likely to report less satisfaction and happiness with their life, perhaps due to their greater negative affect in response to stressors [47, 48]. On the other hand, those with higher aesthetic sensitivity, marked by greater attention to detail and deriving more pleasure from positive experiences, appear to also possess other qualities that denote psychological wellbeing (e.g., curiosity, hopefulness, grit, ambition), which may underlie their enhanced functioning in the absence of adversity and or in positive contexts. The multi-layered associations are in line with previous research, showing that the overall genetic influences on sensitivity consist of multiple components that reflect positive as well as negative sensitivities to the environments [5].

Like previous research with adults, we found that in our sample of adolescents, sensitivity was positively associated with autistic traits. The results suggested that aetiological factors underlying variations in sensitivity also explain variations in autistic traits such as attention to detail and social communication problems. While twin analyses are not equipped to examine the specific underlying mechanisms, it is possible that being generally more sensitive to one’s context increases the risk for social communication problems. In support of this, previous research has found that greater sensitivity is associated with higher introversion and social anxiety [5, 49], and also that hypersensitivities are correlated with social communication difficulties in autistic individuals [50, 51]. Being highly sensitive to one’s physical and psychological context may increase the likelihood of being more easily overwhelmed by stimulation, especially in social interactions when multiple stimuli are competing for processing [6]. Repeated unpleasant overstimulation may therefore lead to avoidance of social situations and subsequently less opportunity to learn to become more competent at them, creating a vicious cycle that increases social communication difficulties.

While we have interpreted our results, in light of previous research, to suggest how genetic influences on sensitivity may relate to variations in autistic traits, we have to emphasise that this does not necessarily mean that genetic sensitivity *causally influences* risk for neurodevelopmental disorders such as autism. It is also possible that these genetic influences contribute to development of both traits, somewhat independently of each other (i.e., horizontal pleiotropy), rather than through each other (i.e., vertical pleiotropy). While the latter process implies a mediation/moderation role for genetics of sensitivity in autistic traits (or vice versa), for example, through causal paths e.g., Oginni, Jern [52], the former does not, and twin analyses conducted herein cannot distinguish between these two potential processes.

### Strength and limitations

The main strength of the current study was the use of a large twin sample to estimate for the first time, the extent to which genetics of environmental sensitivity is implicated in variations in emotional problems, autistic traits and wellbeing. We must however acknowledge certain caveats. First is the limitation in generalisability of the findings from the current study. This is because estimates reflect the heritability of the instrument via which a phenotype is measured, in that specific population, at that specific time. Therefore, the heritability estimates and the genetic correlations obtained in the current study may differ depending on the specific measure that is used across different studies in different populations. Specifically, the current study used an abbreviated self-report measure of sensitivity developed for use in children and adolescents, which has been noted to have a greater emphasis on negative aspects of sensitivity [7, 53]. The relatively low proportion of items about positive aspects of sensitivity may mean that the correlations with internalising problems were amplified and those with wellbeing attenuated. Also, the abbreviated Autism Quotient questionnaire is commonly used to measure autistic traits, but there are disagreements on how well it indexes autism [54, 55]. Future studies are therefore encouraged to use alternative measures to corroborate our findings. Second, all data were collected via questionnaires, mainly self-reports. This could have overinflated the phenotypic correlations and heritability estimates. For example, our sensitivity analyses using an alternative measure of anxiety, which was self-report and focused on awareness of symptoms of anxiety found a larger effect than the parent-report version. Future studies, using other measures and sources, such as interviews and other reports would provide indications as to the robustness of the findings in the current study. Third, our outcome measures were symptoms of clinically diagnosed disorders, rather than clinical diagnosis, and therefore the strength of associations between sensitivity and the disorder may differ and should be examined in future research. Lastly, we were unable to examine longitudinal associations between variables and outcomes, which confounds our interpretation of the direction of effect between variables.

### Future studies

Notwithstanding these limitations, the results of the current study are encouraging for researchers interested in individual differences in risk for psychopathology and the genetic factors that influence susceptibility to mental health problems and wellbeing. The findings call for future studies of environmental sensitivity that aim to better understand its molecular genetic basis. There are currently no genome-wide association studies of environmental sensitivity, despite the potential to collect large-scale data on this phenotype using the available psychometrically validated measures. Future studies could also examine how genetic influences underlying sensitivity interact with various environmental factors to influence the outcomes of such exposures, providing further knowledge of the underlying biological pathways involved in environmental sensitivity and mental health.

In conclusion, the evidence from the current study suggested that genetic factors underlying environmental sensitivity are relevant to explaining individual differences in anxiety and depressive symptoms, autistic traits, and wellbeing. Future genomic studies of environmental sensitivity are encouraged, in order to better understand the heterogeneity in mental health and wellbeing.

## Supplementary information

supplemental materials

## Data Availability

The datasets generated during and/or analysed during the current study are not publicly available as the consent given by the participants does not allow for data storage on an individual level in repositories or journals. Access to data sets requires approval from the relevant access committees at Twins Early Development study (TEDS). Researchers who would like to access the data sets used herein should contact the lead or corresponding authors.

## Code availability

Please contact authors for code availability.
