# Supplementary material for: Genetics of environmental sensitivity and its association with variations in emotional problems, autistic traits, and wellbeing
Source: Mol Psychiatry. 2024 Mar 18;29(8):2438–46. doi: 10.1038/s41380-024-02508-6 (PMC11412899; doi:10.1038/s41380-024-02508-6)
Supplement: Supplementary file 1 — supplemental materials [file 41380_2024_2508_MOESM1_ESM.docx]

**Supplementary Information**

**Title:**  Genetics of environmental sensitivity and its association with variations in emotional problems, autistic traits, and wellbeing

**Study Sample**

The data used in the current study were drawn from three studies in TEDS when twins were approximately 16 years old: Web study, Behaviour/LEAP study and LEAP2 study. Data for wellbeing measures came from the web study, data for autistic traits, depression symptoms and anxiety symptom came from Behaviour/LEAP study, and data for sensitivity came from LEAP2 study.

Briefly, the web study was administered to two cohorts of the total TEDS sample, when twin ages ranged from roughly 16 to 17 during data collection. The behaviour/LEAP study data was collected in two waves and included all cohorts in TEDS. Twin ages ranged from roughly 15 up to 17.5 when booklets were returned. The Behaviour/LEAP study followed directly after the web study in these cohorts; hence the booklet study had a slightly smaller sample because some families had withdrawn, became address problems or became special cases during the web study. Furthermore, some special case and medical exclusion families were included in the web study but not the booklet study, or vice versa, depending on individual circumstances.

The families that were contacted to participate in LEAP-2 study were not contacted at random. Selection for LEAP-2 consisted of families in which one or both twins had scored highly in measures of psychotic experiences in the LEAP study (1420 pairs) and 'control' families in which neither twin had high scores in these measures (355 pairs). Of the families invited, 1,471 (83%) returned the LEAP-2 booklets. Families with LEAP-2 data were more ethnically diverse and included a higher proportion of female twins than those who were not invited to or did not complete the LEAP-2 study, but they did not differ on family socioeconomic characteristics or in the proportion of twins who reported a life event (Peel et al., 2023). The data was collected using paper questionnaires which were sent to the twins for completion. Sample sizes for all variables are presented in Table S2a.

**Wellbeing measures**

The following 8 measures were included to index wellbeing. Table S1a shows descriptive statistics of each variable and Table S1b shows the bi-variate correlations among them.

***Ambition*** was assessed with the ambition subscale of grit and passion for long term goals (Duckworth, Peterson, Matthews, & Kelly, 2007) consisting of 5 items. An example item is “I am driven to succeed”, rated as 1= very much like me, to 5=not like me at all.

***Curiosity*** was measured by the curiosity and exploration self-report questionnaire (Kashdan, Rose, & Fincham, 2004) consisting of 7 items. The total score is a combination of two subscales of exploration and flow, reflected in items such as “I would describe myself as someone who actively seeks as much information as I can in a new situation” and “When I am participating in an activity, I tend to get so involved that I lose track of time”. The items are rated on a scale of 1= strongly agree to 7= strongly disagree.

***Gratitude*** was measured by a self-report questionnaire measuring gratitude via 6 items (McCullough, Emmons, & Tsang, 2002) rated on scale of 1=all of the tome to 7=none of the time. An example item is “As I get older, I find myself more able to appreciate the people, events, and situations that have been part of my life history”.

***Grit*** was assessed with the short Grit scale (Duckworth & Quinn, 2009) consisting of 8 items, measuring grit and perseverance, reflected in two subscales consistently of interest and perseverance. An example item is “Setbacks don’t discourage me”, rated as 1= very much like me, to 5=not like me at all.

***Happiness*** was assessed with the Subjective Happiness scale (Lyubomirsky & Lepper, 1999), a self-report questionnaire consisting of 4 items that measures how individuals describe themselves in terms of how happy they are. An example item is “Some people are generally very happy. They enjoy life regardless of what is going on, getting the most out of everything. To what extent does this describe you?”, rated on a scale of 1=not at all to 7=a great deal.

***Hopefulness*** was measured by the Children’s Hope scale (Snyder et al., 1997), consisting of 6 items. An example item is “Even when others want to quit, I know that I can find ways to solve the problem” rated on a 6-point Lickert scale (1=all of the time, 6=none of the times).

***Life satisfaction*** was assessed with the Brief Multidimensional Life Satisfaction scale (Seligson, Huebner, & Valois, 2003), comprising 21 items, asking participants to rate their satisfaction with various aspects of their life on a 6-point Likert scale (1=strongly agree , 6=strongly disagree). The total score of life satisfaction reflects five subscales that include family, life, school, living environment and life satisfaction.

***Optimism*** was assessed with the revised Life Orientation Test (Scheier, Carver, & Bridges, 1994) . The self-report questionnaire consists of a total of 6 items, three items for optimism, and three for pessimism. Participants rate their agreement with the items on a 5-point Likert scale (1 = strongly disagree, 5 = strongly agree). An example item is “In uncertain times, I usually expect the best”.

| **Table S1a**. Descriptive statistics of wellbeing measures | | | | | | | | | |
| --- | --- | --- | --- | --- | --- | --- | --- | --- | --- |
|  | N | mean | sd | min | max | range | skew | kurtosis | se |
| Hopefulness | 5442 | 4.7 | 0.73 | 1 | 6 | 5 | -0.88 | 1.52 | 0.01 |
| Curiosity | 5430 | 4.79 | 0.91 | 1.14 | 7 | 5.86 | -0.2 | -0.06 | 0.01 |
| Ambition | 4748 | 3.91 | 0.68 | 1 | 5 | 4 | -0.49 | 0.09 | 0.01 |
| Grit | 4841 | 3.27 | 0.59 | 1 | 5 | 4 | 0.01 | 0.52 | 0.01 |
| Optimism | 4842 | 3.23 | 0.73 | 1 | 5 | 4 | -0.25 | 0.17 | 0.01 |
| Happiness | 9932 | 5.11 | 0.96 | 1 | 7 | 6 | -0.52 | 0.38 | 0.01 |
| Gratitude | 5442 | 5.79 | 0.85 | 1 | 7 | 6 | -0.8 | 0.71 | 0.01 |
| Life Satisfaction | 9928 | 5.7 | 1.06 | 1 | 7 | 6 | -1.12 | 1.06 | 0.01 |

| **Table S1b**. Correlations between wellbeing measures | | | | | | | |
| --- | --- | --- | --- | --- | --- | --- | --- |
|  | Hopefulness | Optimism | Happiness | Curiosity | Gratitude | Ambition | Grit |
| Optimism | .46* |  |  |  |  |  |  |
| Happiness | .45* | .48* |  |  |  |  |  |
| Curiosity | .50* | .21** | .23* |  |  |  |  |
| Gratitude | .51* | .37* | .40* | .34* |  |  |  |
| Ambition | .50* | .34* | .22* | .51* | .41* |  |  |
| Grit | .36* | .39* | .28* | .21* | .37* | .44* |  |
| Satisfaction | .46* | .45* | .66* | .16* | .43* | .24* | .30* |
|  | | | | | | | |

**Principal Component analysis**

Principal component analyses were conducted on 8 measures of wellbeing that included life satisfaction, Grit, Ambition, Gratitude, Curiosity, subjective happiness, Optimism and Hopefulness. The sample included all twins in TEDS who contributed to data when they were approximately 16 years old (N ranging from 5k to 9k depending on the measure, see Table S1a). Data analysis was restricted to participants with complete data on all of the eight wellbeing measures (N=3,796). Principal components were extracted based on eigenvalues > 1, and direct Oblimin with Kaizer normalisation rotation method, using SPSS version 24.

**
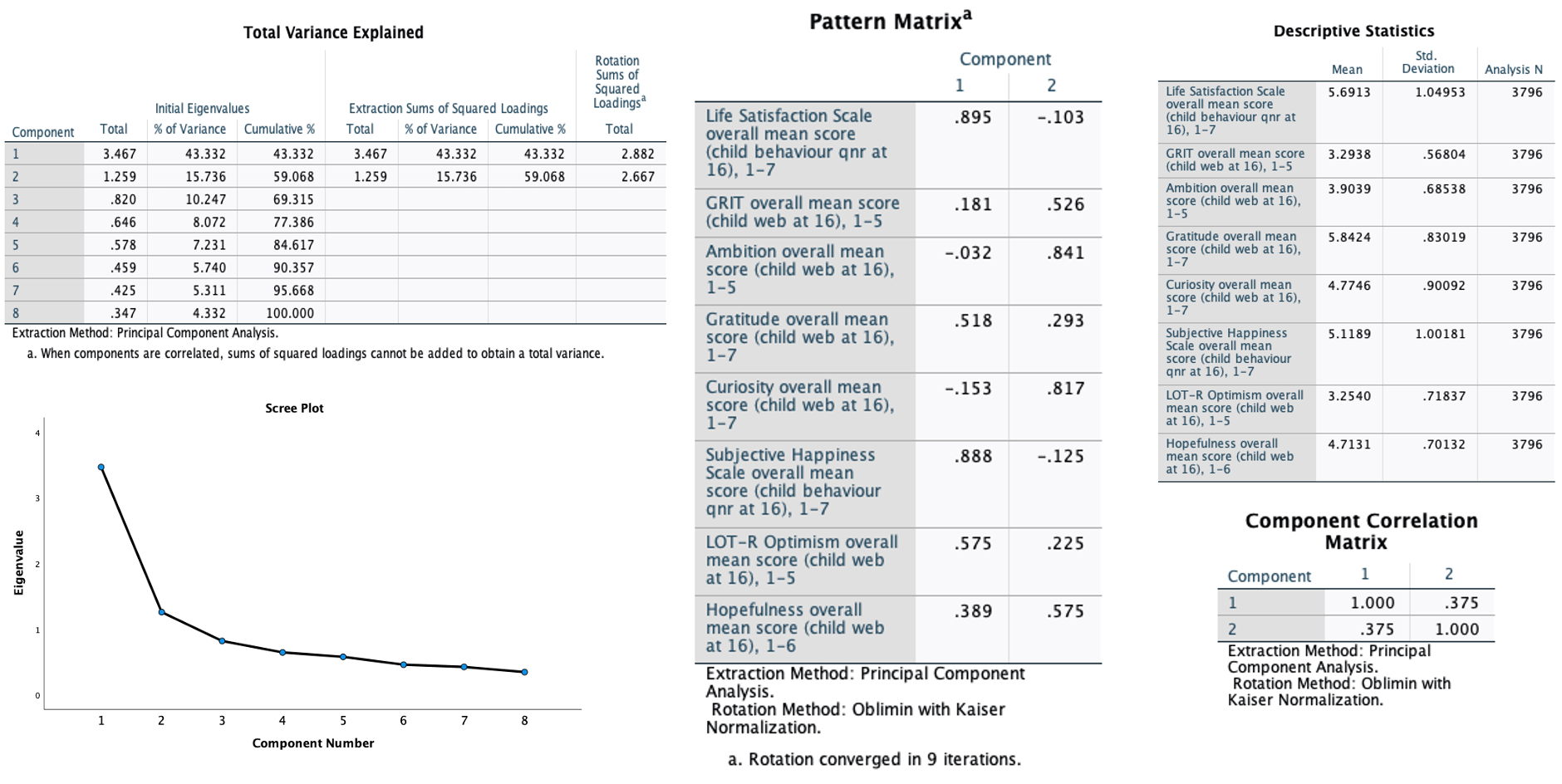
**

**Figure S1.** Principal component analyses on wellbeing measures

**Descriptive statistics**

Sample sizes and descriptive statistics of the variables used in the analyses are presented in Table S2a. Bivariate correlations between all variables are presented in Table S2b.

Analysis of variance by sensitivity group is presented in Tables S3a-S3c.

| **Table S2a.** Descriptive statistics of the sample and data used in the current study | | | | | | | | | | | |
| --- | --- | --- | --- | --- | --- | --- | --- | --- | --- | --- | --- |
|  | Sample descriptives | | |  | Data descriptives | | | | | | |
|  | N | Age (sd)* | male/female |  | mean score (sd) | min | max | range | skew | kurtosis | se |
| Sensitivity | 2944 | 17.06 (.88) | 1072/1242 |  | 3.98 (.94) | 1 | 7 | 6 | .02 | -.19 | .02 |
| Aesthetic | 2944 | 17.06 (.88) | 1072/1243 |  | 5.17 (.99) | 1 | 7 | 6 | -.74 | .87 | .02 |
| Excitation | 2944 | 17.06 (.88) | 1072/1244 |  | 3.81 (1.36) | 1 | 7 | 6 | .00 | -.62 | .03 |
| Sensory | 2944 | 17.06 (.88) | 1072/1245 |  | 2.68 (1.34) | 1 | 7 | 6 | .68 | -.22 | .02 |
| Anxiety symptoms (self-report) ** | 2942 | 16.30 (.68) | 1072/1240 |  | 8.97 (6.54) | 0 | 35 | 35 | 1.04 | .86 | .12 |
| Anxiety symptoms (parent-report)** | 2944 | 16.30 (.68) | 1072/1242 |  | 4.42 (5.02) | 0 | 33 | 33 | 1.76 | 3.53 | .09 |
| Depression symptoms** | 2941 | 16.30 (.68) | 1700/1241 |  | 4.82 (5.42) | 0 | 26 | 26 | 1.52 | 1.97 | .1 |
| Autistic traits | 2940 | 16.30 (.68) | 1700/1240 |  | 12.75 (6.28) | 0 | 39 | 39 | .45 | -.01 | .12 |
| Subjective wellbeing | 1163 | 16.46 (.26) | 723/440 |  | -.20 (1.14) | -4.90 | 2.14 | 7.04 | -.63 | .26 | .03 |
| Psychological wellbeing | 1163 | 16.46 (.26) | 723/440 |  | -.01 (1.09) | -4.24 | 2.87 | 7.11 | -.33 | .07 | .03 |
| * Age of twin at the time of data collection; Sensitivity= sensitivity total score; Aesthetic= Aesthetic sensitivity factor; Excitation=Excitation sensitivity factor; Sensory= Sensory sensitivity factor; Anxiety symptoms (parent-report)=Anxiety Related Behaviours Questionnaire; Anxiety symptoms (self-report) =self-report Child Anxiety Sensitivity Index **statistics reflect distribution of data prior to rank transformation for subsequent twin analyses. | | | | | | | | | | | |

| **Table S2b. correlations between all study variables** | | | | | | | | | | | | | | | | | | |
| --- | --- | --- | --- | --- | --- | --- | --- | --- | --- | --- | --- | --- | --- | --- | --- | --- | --- | --- |
|  |  | Sens | 1 | 2 | 3 | 4 | 5 | 6 | 7 | 8 | 9 | 10 | 11 | 12 | 13 | 14 | 15 | 16 |
| 1 | Aesthetic | .58* |  |  |  |  |  |  |  |  |  |  |  |  |  |  |  |  |
| 2 | Excitation | .89* | .28* |  |  |  |  |  |  |  |  |  |  |  |  |  |  |  |
| 3 | Sensory | .74* | .18* | .54* |  |  |  |  |  |  |  |  |  |  |  |  |  |  |
| 4 | Hope | -.19* | .15* | -.30* | -.13* |  |  |  |  |  |  |  |  |  |  |  |  |  |
| 5 | Optimism | -.21* | .10* | -.32* | -.14* | .46* |  |  |  |  |  |  |  |  |  |  |  |  |
| 6 | Happiness | -.22* | .08* | -.31* | -.18* | .45* | .48* |  |  |  |  |  |  |  |  |  |  |  |
| 7 | Curiosity | -.01 | .24* | -.13* | -.02 | .50* | .21** | .23* |  |  |  |  |  |  |  |  |  |  |
| 8 | Gratitude | -.07* | .18* | -.18* | -.06* | .51* | .37* | .40* | .34* |  |  |  |  |  |  |  |  |  |
| 9 | Ambition | -.04 | .22* | -.16* | -.04 | .50* | .34* | .22* | .51* | .41* |  |  |  |  |  |  |  |  |
| 10 | Grit | -.19* | .07* | -.29* | -.11* | .36* | .39* | .28* | .21* | .37* | .44* |  |  |  |  |  |  |  |
| 11 | Satisfaction | -.29* | .02 | -.36* | -.22* | .46* | .45* | .66* | .16* | .43* | .24* | .30* |  |  |  |  |  |  |
| 12 | Autistic traits | .32* | .04 | .34* | .30* | -.19* | -.27* | -.38* | .02 | -.27* | -.09* | -.14* | -.38* |  |  |  |  |  |
| 13 | Depressive | .35* | .07 | .38* | .25* | -.36* | -.38* | -.57* | -.07* | -.23* | -.13* | -.24* | -.65* | .32* |  |  |  |  |
| 14 | Anxiety  (self-report) | .46* | .19* | .45* | .34* | -.23* | -.23* | -.30* | -.01 | -.07* | .01 | -.14* | -.39* | .30* | .54* |  |  |  |
| 15 | Anxiety  (parent-report) | .21* | -.02 | .25* | .19* | -.28* | -.21* | -.31* | -.14* | -.18* | -.12* | -.08* | -.30* | .31* | .29* | .23* |  |  |
| 16 | Subj. well | -.32* | .09* | -.42* | -.24* | .63* | .70* | .87* | .24* | .63* | .32* | .44* | .87* | -.44* | -.65* | -.37* | -.31* |  |
| 17 | Psych. well | -.09* | .24* | -.24* | -.06* | .74* | .46* | .27* | .78* | .57* | .84* | .60* | .27* | -.09* | -.17* | -.05 | -.18* | .43* |
| Sens= sensitivity total score; Aesthetic= Aesthetic sensitivity factor; Excitation=Excitation sensitivity factor; Sensory= Sensory sensitivity factor; Hope=Hopefulness; Sat=satisfaction; Depressive=depression symptoms; Anxiety(parent-report)= anxiety symptoms from anxiety related behaviours questionnaire; Anxiety(self-report)= anxiety symptoms from Child Anxiety sensitivity index; Subj. well= subjective wellbeing factor score; Psych. well= psychological wellbeing factor score | | | | | | | | | | | | | | | | | | |

| **Table S3a. Mean and variances in low vs. high sensitivity groups (25th vs. 7th percentile scores)** | | | | | | | | | |
| --- | --- | --- | --- | --- | --- | --- | --- | --- | --- |
| sensitivity score | | Anxiety (ARBQ) | Anxiety | Depression | Autistic traits | Hopefulness | Optimism | Happiness | Satisfaction |
|  |  |  | (CASI) |  |  |  |  |  |  |
| 25th percentile | Mean | 3.23 | 5.36 | 2.63 | 10.27 | 4.83 | 3.35 | 5.28 | 5.9 |
|  | N | 705 | 703 | 704 | 704 | 285 | 274 | 704 | 703 |
|  | SD | 4.1 | 4.84 | 4.03 | 5.55 | 0.74 | 0.75 | 1 | 1.08 |
|  | Variance | 16.78 | 23.42 | 16.26 | 30.81 | 0.54 | 0.56 | 1 | 1.16 |
| 75th percentile | Mean | 6.13 | 13.61 | 7.68 | 15.35 | 4.39 | 2.94 | 4.62 | 4.98 |
|  | N | 367 | 367 | 367 | 365 | 161 | 148 | 366 | 365 |
|  | SD | 5.77 | 7.36 | 6.5 | 6.3 | 0.88 | 0.83 | 1.2 | 1.26 |
|  | Variance | 33.34 | 54.2 | 42.28 | 39.74 | 0.77 | 0.68 | 1.44 | 1.59 |

| **Table S3b. Leven’s test of homogeneity of variance: low vs. high sensitivity groups (25th vs. 7th percentile scores)** | | | | | |
| --- | --- | --- | --- | --- | --- |
|  | | Levene Statistic | df1 | df2 | Sig. |
| Anxiety (ARBQ) | Based on Mean | 61.962 | 1 | 1070 | **<.001** |
|  | Based on Median | 50.362 | 1 | 1070 | **<.001** |
|  | Based on Median and with adjusted df | 50.362 | 1 | 1035.89 | **<.001** |
|  | Based on trimmed mean | 57.874 | 1 | 1070 | **<.001** |
| Anxiety (CASI) | Based on Mean | 111.514 | 1 | 1068 | **<.001** |
|  | Based on Median | 103.993 | 1 | 1068 | **<.001** |
|  | Based on Median and with adjusted df | 103.993 | 1 | 1041.93 | **<.001** |
|  | Based on trimmed mean | 112.282 | 1 | 1068 | **<.001** |
| Depression | Based on Mean | 147.506 | 1 | 1069 | **<.001** |
|  | Based on Median | 117.623 | 1 | 1069 | **<.001** |
|  | Based on Median and with adjusted df | 117.623 | 1 | 1035.93 | **<.001** |
|  | Based on trimmed mean | 146.148 | 1 | 1069 | **<.001** |
| Autistic traits | Based on Mean | 5.088 | 1 | 1067 | **0.024** |
|  | Based on Median | 5.107 | 1 | 1067 | **0.024** |
|  | Based on Median and with adjusted df | 5.107 | 1 | 1042.29 | **0.024** |
|  | Based on trimmed mean | 5.278 | 1 | 1067 | **0.022** |
| Hopefulness | Based on Mean | 6.768 | 1 | 444 | **0.010** |
|  | Based on Median | 4.028 | 1 | 444 | **0.045** |
|  | Based on Median and with adjusted df | 4.028 | 1 | 426.13 | **0.045** |
|  | Based on trimmed mean | 5.817 | 1 | 444 | **0.016** |
| Optimism | Based on Mean | 2.674 | 1 | 420 | 0.103 |
|  | Based on Median | 2.580 | 1 | 420 | 0.109 |
|  | Based on Median and with adjusted df | 2.580 | 1 | 418.79 | 0.109 |
|  | Based on trimmed mean | 2.600 | 1 | 420 | 0.108 |
| Happiness | Based on Mean | 17.559 | 1 | 1068 | **<.001** |
|  | Based on Median | 16.167 | 1 | 1068 | **<.001** |
|  | Based on Median and with adjusted df | 16.167 | 1 | 1035.08 | **<.001** |
|  | Based on trimmed mean | 17.160 | 1 | 1068 | **<.001** |
| Satisfaction | Based on Mean | 16.731 | 1 | 1066 | **<.001** |
|  | Based on Median | 16.986 | 1 | 1066 | **<.001** |
|  | Based on Median and with adjusted df | 16.986 | 1 | 1065.68 | **<.001** |
|  | Based on trimmed mean | 18.412 | 1 | 1066 | **<.001** |

| **Table S3c. ANNOVA comparing high vs low sensitivity scores** | | | | | | | |
| --- | --- | --- | --- | --- | --- | --- | --- |
|  | | | Sum of Squares | df | Mean Square | F | Sig. |
| Anxiety (ARBQ) | Between Groups | (Combined) | 2035.16 | 1 | 2035.16 | 90.68 | **<.001** |
|  | Within Groups | | 24013.68 | 1070 | 22.44 |  |  |
|  | Total | | 26048.84 | 1071 |  |  |  |
| Anxiety (CASI) | Between Groups | (Combined) | 16428.13 | 1 | 16428.13 | 483.65 | **<.001** |
|  | Within Groups | | 36276.42 | 1068 | 33.97 |  |  |
|  | Total | | 52704.55 | 1069 |  |  |  |
| Depression | Between Groups | (Combined) | 6129.56 | 1 | 6129.56 | 243.53 | **<.001** |
|  | Within Groups | | 26905.80 | 1069 | 25.17 |  |  |
|  | Total | | 33035.36 | 1070 |  |  |  |
| Autistic traits | Between Groups | (Combined) | 6217.94 | 1 | 6217.94 | 183.67 | **<.001** |
|  | Within Groups | | 36121.69 | 1067 | 33.85 |  |  |
|  | Total | | 42339.63 | 1068 |  |  |  |
| Hopefulness | Between Groups | (Combined) | 19.43 | 1 | 19.43 | 31.13 | **<.001** |
|  | Within Groups | | 277.11 | 444 | 0.62 |  |  |
|  | Total | | 296.54 | 445 |  |  |  |
| Optimism | Between Groups | (Combined) | 15.92 | 1 | 15.92 | 26.41 | **<.001** |
|  | Within Groups | | 253.15 | 420 | 0.60 |  |  |
|  | Total | | 269.07 | 421 |  |  |  |
| Happiness | Between Groups | (Combined) | 104.80 | 1 | 104.80 | 91.40 | **<.001** |
|  | Within Groups | | 1224.62 | 1068 | 1.15 |  |  |
|  | Total | | 1329.42 | 1069 |  |  |  |
| Satisfaction | Between Groups | (Combined) | 205.28 | 1 | 205.28 | 157.31 | **<.001** |
|  | Within Groups | | 1391.02 | 1066 | 1.30 |  |  |
|  | Total | | 1596.30 | 1067 |  |  |  |

**Multivariate twin analyses**

**Model 1:** correlated factors solution of the Cholesky decomposition model

To examine aim 1, a correlated factors solution of a multivariate Cholesky decomposition model was constructed. This is a variation of the Cholesky model whereby the genetic and environmental paths between variables are interpreted as genetic and environmental correlations. Model fit results are presented in Table S4.

| \| **Table S4**. Model 1 (correlated factors solution of the Cholesky decomposition model) results for sensitivity, anxiety and depressive symptoms, autistic traits, and wellbeing \| \| --- \| | | | | | | | | |
| --- | --- | --- | --- | --- | --- | --- | --- | --- | --- |
| **base** | **comparison** | **ep** | **minus2LL** | **df** | **AIC** | **Δ -2ll** | **Δ df** | **p** |
| Sat |  | 130 | 130012.10 | 36392 | 130272.10 |  |  |  |
| Sat | Sub | 50 | 130107.30 | 36472 | 130207.30 | 95.26 | 80 | 0.12 |
| Sub |  | 50 | 130107.30 | 36472 | 130207.30 |  |  |  |
| Sub | ACE | 50 | 130129.00 | 36472 | 130229.00 | 21.61 | 0 | *NA* |
| Sub | AE245 | 41 | 130135.60 | 36481 | 130217.60 | 28.24 | 9 | 9E-04 |
| Sub | AE24 | 38 | 130143.40 | 36484 | 130219.40 | 36.08 | 12 | 3E-04 |
| Sub | AE2 | 36 | 130145.10 | 36486 | 130217.10 | 37.78 | 14 | 6E-04 |
| Sub | AE | 35 | 130184.40 | 36487 | 130254.40 | 77.04 | 15 | 2E-10 |
| ACE |  | 50 | 130129.00 | 36472 | 130229.00 |  |  |  |
| ACE | AE245 | 41 | 130135.60 | 36481 | 130217.60 | 6.63 | 9 | 0.68 |
| ACE | AE24 | 38 | 130143.40 | 36484 | 130219.40 | 14.47 | 12 | 0.27 |
| ACE | AE2 | 36 | 130145.10 | 36486 | 130217.10 | 16.17 | 14 | 0.30 |
| ACE | AE | 35 | 130184.40 | 36487 | 130254.40 | 55.43 | 15 | 2E-06 |
| Sat= Saturated model with maximum number of parameters describing the data; Sub=constrained sub-model of the fully saturated model, testing the assumptions of twin design, with means and variances equated across twins and zygosity; ACE= univariate ACE model; AE245: submodel of the ACE model where only the C paths for variables 2, 4 and 5 were kept in the model and non-significant C paths were dropped for other variables. AE24: submodel of the ACE245 model where the C path for variable 5 is dropped; AE2: submodel of the ACE24 model where only the C paths for variable 4 is dropped; AE= submodel of the ACE2 model where all C paths are dropped; −2ll= minus twice the log likelihood; df= degrees of freedom; AIC= Akaike’s information criterion; Δ -2ll =difference in -2ll value; Δ df= difference in degrees of freedom; p= p-value; The best fitting models are marked as bold, selected based on the principle of parsimony and lowest AIC and -2ll value. A difference in AIC between two models of 2 or less, provides equivalent support for both models, in which case the most parsimonious model (i.e. with lowest number of parameters) was chosen, a difference of 3 indicates that the lower AIC model has considerably more support, and a difference of more than 10, indicates that the lower AIC model is a substantially better fit compared to the higher AIC model. | | | | | | | | |

**Sensitivity analyses: Model 1**

Two sensitivity analyses were run for Model 1. In the first, the parent-report measure of anxiety in the model was replaced with a self-report measure, the Childhood Anxiety Sensitivity Index (CASI; Silverman, Fleisig, Rabian, & Peterson, 1991). This measure mainly captures adolescents’ awareness of, and tendency to, negatively interpret symptoms of anxiety (Chorpita, Albano, & Barlow, 1996). Second, the total environmental sensitivity score was replaced with “Excitation” and “Sensory” factors in the model to examine their association with mental health outcomes and wellbeing. The Aesthetic factor model was run only as a bivariate model with psychological wellbeing, since none of the other correlations surpassed the r >.2 threshold (see Table S2b).

| **Table S5a**. Model 1 (correlated factors solution of the Cholesky decomposition model) fit results using **CASI for anxiety symptoms** | | | | | | | | |
| --- | --- | --- | --- | --- | --- | --- | --- | --- |
| **base** | **comparison** | **ep** | **minus2LL** | **df** | **AIC** | **Δ -2ll** | **Δ df** | **p** |
| Sat |  | 130 | 129189.50 | 36347 | 129449.50 |  |  |  |
| Sat | Sub | 50 | 129282.50 | 36427 | 129382.50 | 92.95 | 80 | 0.15 |
| Sub |  | 50 | 129282.50 | 36427 | 129382.50 |  |  |  |
| Sub | ACE | 50 | 129305.80 | 36427 | 129405.80 | 23.35 | 0 | *NA* |
| Sub | AE245 | 41 | 129313.80 | 36436 | 129395.80 | 31.36 | 9 | 3E-04 |
| Sub | AE24 | 38 | 129320.60 | 36439 | 129396.60 | 38.17 | 12 | 1E-04 |
| Sub | AE2 | 36 | 129322.20 | 36441 | 129394.20 | 39.78 | 14 | 3E-04 |
| Sub | AE | 35 | 129322.30 | 36442 | 129392.30 | 39.86 | 15 | 5E-04 |
| ACE |  | 50 | 129305.80 | 36427 | 129405.80 |  |  |  |
| ACE | AE245 | 41 | 129313.80 | 36436 | 129395.80 | 8.01 | 9 | 0.53 |
| ACE | AE24 | 38 | 129320.60 | 36439 | 129396.60 | 14.82 | 12 | 0.25 |
| ACE | AE2 | 36 | 129322.20 | 36441 | 129394.20 | 16.42 | 14 | 0.29 |
| **ACE** | **AE** | **35** | **129322.30** | **36442** | **129392.30** | **16.50** | **15** | **0.35** |
| Sat= Saturated model with maximum number of parameters describing the data; Sub=constrained sub-model of the fully saturated model, testing the assumptions of twin design, with means and variances equated across twins and zygosity; ACE= univariate ACE model; AE245: sub-model of the ACE model where only the C paths for variables 2, 4 and 5 were kept in the model and non-significant C paths were dropped for other variables. AE24: sub-model of the ACE245 model where the C path for variable 5 is dropped; AE2: sub-model of the ACE24 model where the C paths for variable 4 is dropped; AE= sub-model of the ACE2 model where all C paths are dropped; −2ll= minus twice the log likelihood; df= degrees of freedom; AIC= Akaike’s information criterion; Δ -2ll =difference in -2ll value; Δ df= difference in degrees of freedom; p= p-value; The best fitting models are marked as bold, selected based on the principle of parsimony and lowest AIC and -2ll value. A difference in AIC between two models of 2 or less, provides equivalent support for both models, in which case the most parsimonious model (i.e. with lowest number of parameters) was chosen, a difference of 3 indicates that the lower AIC model has considerably more support, and a difference of more than 10, indicates that the lower AIC model is a substantially better fit compared to the higher AIC model. | | | | | | | | |


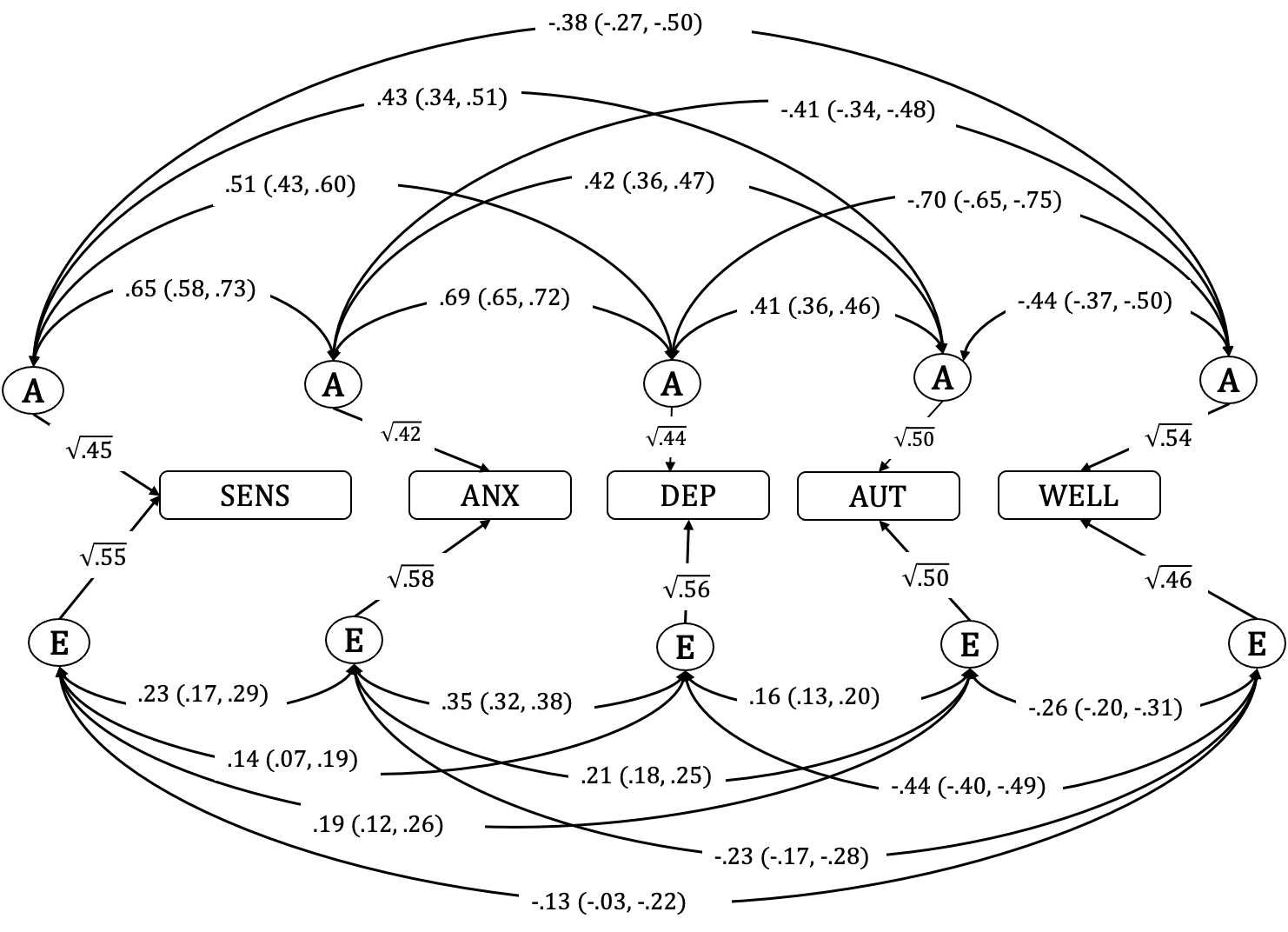


**Figure S2.** Model 1 (correlated factors solution of the Cholesky decomposition model) using **CASI for anxiety symptoms**. ANX=anxiety symptoms; AUT=autistic traits; DEP=depressive symptoms; WELL=subjective wellbeing factor; A= additive genetic influences; C= common environmental influences; E= unique environmental influences. 95% Confidence intervals are shown in parentheses. Straight arrows represent contributions from ACE components to each variable, and curved arrows represent correlations between AE components for variables.

| **Table S5b**. Model 1 (correlated factors solution of the Cholesky decomposition model) estimates using **CASI for** **anxiety symptoms** | | | | | | |
| --- | --- | --- | --- | --- | --- | --- |
|  |  | **rPh** | **rPhA** | **rphE** | **% rPhA** | **% rPhE** |
| **Sensitivity** | Anx | .41 (.38, .44) | .28 (.24, .31) | .13 (.09, .17) | 68 | 32 |
|  | Dep | .30 (.27, .33) | .23 (.20, .27) | .08 (.04, .11) | 77 | 27 |
|  | AUT | .31 (.28, .34) | .20 (.16, .24) | .10 (.06, .14) | 65 | 32 |
|  | Well | -.25 (-.30, -.21) | -.19 (-.24, -.13) | -.06 (-.11, -.02) | 76 | 24 |
| **Anxiety symptoms** | Dep | .50 (.48, .51) | .30 (.27, .32) | .20 (.18, 0.22) | 60 | 40 |
|  | AUT | .30 (.29, .32) | .19 (.17, .22) | .11 (.09, .13) | 63 | 37 |
|  | Well | -.31 (-.34, -.29) | -.19 (-.23, -.16) | -.12 (-.15, -.09) | 61 | 39 |
| **Depressive symptoms** | AUT | .28 (.26, .30) | .19 (.17, .22) | .09 (.07, .11) | 68 | 32 |
|  | Well | -.57 (-.59, -.55) | -.34 (-.37, -.31) | -.23 (-.25, -.20) | 60 | 40 |
| **Autistic traits** | Well | -.35 (-.38, -.33) | -.23 (-.26, -.19) | -.12 (-.15, -.10) | 66 | 34 |
| Results from the best fitting AE model; Anx=anxiety symptoms; Dep=depressive symptoms; AUT= autistic traits; Well= subjective wellbeing; rPh= phenotypic correlation; rPhA= phenotypic correlation due to correlating genetic effects; rPhE= phenotypic correlation due to correlating environmental effects; % rPhA= percentage of phenotypic correlation due to correlating genetic effects; % rPhE= percentage of phenotypic correlation due to correlating environmental effects; Percentages are rounded up | | | | | | |

| **Table S6a**. Model1 (correlated factors solution of the Cholesky decomposition model) fit results for **Excitation sensitivity factor** | | | | | | | | |
| --- | --- | --- | --- | --- | --- | --- | --- | --- |
| **base** | **comparison** | **ep** | **minus2LL** | **df** | **AIC** | **Δ -2ll** | **Δ df** | **p** |
| Sat |  | 130 | 132098.70 | 36392 | 132358.70 |  |  |  |
| Sat | Sub | 50 | 132194.70 | 36472 | 132294.70 | 95.97 | 80 | 0.11 |
| Sub |  | 50 | 132194.70 | 36472 | 132294.70 |  |  |  |
| Sub | ACE | 50 | 132214.90 | 36472 | 132314.90 | 20.24 | 0 | *NA* |
| Sub | AE245 | 41 | 132221.00 | 36481 | 132303.00 | 26.30 | 9 | 2E-03 |
| Sub | AE24 | 38 | 132228.30 | 36484 | 132304.30 | 33.63 | 12 | 8E-04 |
| Sub | AE2 | 36 | 132230.60 | 36486 | 132302.60 | 35.95 | 14 | 1E-03 |
| Sub | AE | 35 | 132269.60 | 36487 | 132339.60 | 74.92 | 15 | 6E-10 |
| ACE |  | 50 | 132214.90 | 36472 | 132314.90 |  |  |  |
| ACE | AE245 | 41 | 132221.00 | 36481 | 132303.00 | 6.06 | 9 | 0.73 |
| ACE | AE24 | 38 | 132228.30 | 36484 | 132304.30 | 13.39 | 12 | 0.34 |
| **ACE** | **AE2** | **36** | **132230.60** | **36486** | **132302.60** | **15.71** | **14** | **0.33** |
| ACE | AE | 35 | 132269.60 | 36487 | 132339.60 | 54.68 | 15 | 2E-06 |
| Sat= Saturated model with maximum number of parameters describing the data; Sub=constrained sub-model of the fully saturated model, testing the assumptions of twin design, with means and variances equated across twins and zygosity; ACE= univariate ACE model; AE245: sub-model of the ACE model where only the C paths for variables 2, 4 and 5 were kept in the model and non-significant C paths were dropped for other variables. AE24: sub-model of the ACE245 model where the C path for variable 5 is dropped; AE2: sub-model of the ACE24 model where the C paths for variable 4 is dropped; AE: sub-model of the ACE2 model where all C paths are dropped;−2ll= minus twice the log likelihood; df= degrees of freedom; AIC= Akaike’s information criterion; Δ -2ll =difference in -2ll value; Δ df= difference in degrees of freedom; p= p-value; The best fitting models are marked as bold, selected based on the principle of parsimony and lowest AIC and -2ll value. A difference in AIC between two models of 2 or less, provides equivalent support for both models, in which case the most parsimonious model (i.e. with lowest number of parameters) was chosen, a difference of 3 indicates that the lower AIC model has considerably more support, and a difference of more than 10, indicates that the lower AIC model is a substantially better fit compared to the higher AIC model. | | | | | | | | |


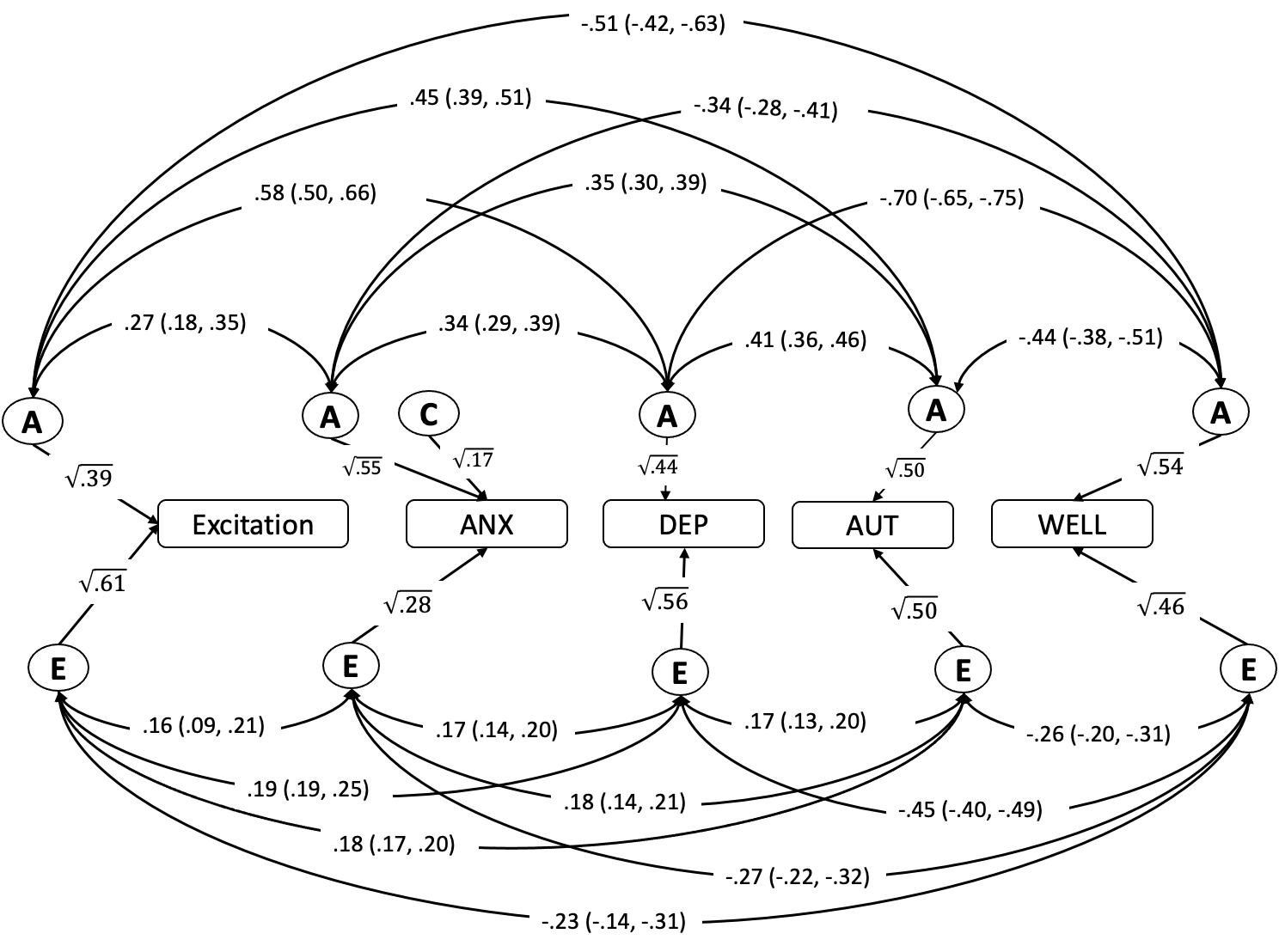


**Figure S3.** Model 1 (correlated factors solution of the Cholesky decomposition model) for **Excitation sensitivity factor.** ANX=anxiety symptoms; AUT=autistic traits; DEP=depressive symptoms; WELL=subjective wellbeing factor; A= additive genetic influences; C= common environmental influences; E= unique environmental influences. 95% Confidence intervals are shown in parentheses. Straight arrows represent contributions from ACE components to each variable, and curved arrows represent correlations between AE components for variables.

| **Table S6b**. Model 1 (correlated factors solution of the Cholesky decomposition model) estimates for **Excitation sensitivity factor** | | | | | | |
| --- | --- | --- | --- | --- | --- | --- |
|  |  | **rPh** | **rPhA** | **rPhE** | **% rPhA** | **% rPhE** |
| **Excitation** |  |  |  |  |  |  |
|  | Anx | .19 (.16, .22) | .13 (.09, .16) | .06 (.04, .09) | 68 | 32 |
|  | Dep | .35 (.33, .38) | .24 (.21, .25) | .11 (.11, .15) | 69 | 31 |
|  | AUT | .30 (.27, .32) | .20 (.17, .23) | .10 (.09, .11) | 67 | 33 |
|  | Well | -.36 (-.39, -.32) | -.24 (-.29, -.18) | -.12 (-.17, -.08) | 67 | 33 |
| **Anxiety symptoms** | Dep | .23 (.21, .25) | .17 (.14, .19) | .07 (.06, .08) | 74 | 26 |
|  | AUT | .25 (.23, .27) | .18 (.16, .20) | .07 (.05, .08) | 72 | 28 |
|  | Well | -.28 (-.31, -.25) | -.19 (-.22, -.15) | -.10 (-.12, -.08) | 68 | 32 |
| **Depressive symptoms** | AUT | .28 (.26, .30) | .19 (.17, .22) | .09 (.07, .11) | 68 | 32 |
|  | Well | -.57 (-.59, -.55) | -.34 (-.37, -.31) | -.23 (-.25, -.20) | 60 | 40 |
| **Autistic traits** | Well | -.35 (-.38, -.33) | -.23 (-.27, -.20) | -.12 (-.15, -.10) | 66 | 34 |
| Results from the best fitting AE model (AE2); Excitation=Excitation sensitivity factor; Anx=anxiety symptoms; Dep=depressive symptoms; AUT= autistic traits; Well=subjective wellbeing; rPh= phenotypic correlation; rPhA= phenotypic correlation due to correlating genetic effects; rPhE= phenotypic correlation due to correlating environmental effects; % rPhA= percentage of phenotypic correlation due to correlating genetic effects; % rPhE= percentage of phenotypic correlation due to correlating environmental effects; Percentages are rounded up | | | | | | |

| **Table S57**. Model 1 (Cholesky correlated factors) fit results for **Sensory sensitivity factor** | | | | | | | | |
| --- | --- | --- | --- | --- | --- | --- | --- | --- |
| **base** | **comparison** | **ep** | **minus2LL** | **df** | **AIC** | **Δ -2ll** | **Δ df** | **p** |
| Sat | | 130 | 132337.30 | 36392 | 132597.30 |  |  |  |
| Sat | Sub | 50 | 132429.00 | 36472 | 132529.00 | 91.69 | 80 | 0.17 |
| Sub | | 50 | 132429.00 | 36472 | 132529.00 |  |  |  |
| Sub | ACE | 50 | 132452.60 | 36472 | 132552.60 | 23.61 | 0 |  |
| Sub | AE245 | 41 | 132459.20 | 36481 | 132541.20 | 30.17 | 9 | 4E-04 |
| Sub | AE24 | 38 | 132467.80 | 36484 | 132543.80 | 38.78 | 12 | 1E-04 |
| Sub | AE2 | 36 | 132469.60 | 36486 | 132541.60 | 40.61 | 14 | 2E-04 |
| Sub | AE | 35 | 132509.40 | 36487 | 132579.40 | 80.41 | 15 | 6E-11 |
| ACE | | 50 | 132452.60 | 36472 | 132552.60 |  |  |  |
| ACE | AE245 | 41 | 132459.20 | 36481 | 132541.20 | 6.56 | 9 | 0.68 |
| ACE | AE24 | 38 | 132467.80 | 36484 | 132543.80 | 15.16 | 12 | 0.23 |
| **ACE** | **AE2** | **36** | **132469.60** | **36486** | **132541.60** | **17.00** | **14** | **0.26** |
| ACE | AE | 35 | 132509.40 | 36487 | 132579.40 | 56.80 | 15 | 9E-07 |
| Sat= Saturated model with maximum number of parameters describing the data; Sub=constrained sub-model of the fully saturated model, testing the assumptions of twin design, with means and variances equated across twins and zygosity; ACE= univariate ACE model; AE245: sub-model of the ACE model where only the C paths for variables 2, 4 and 5 were kept in the model and non-significant C paths were dropped for other variables. AE24: sub-model of the ACE245 model where the C paths for variable 5 is dropped; AE2: sub-model of the ACE24 model where the C paths for variable 4 is dropped; AE: sub-model of the ACE2 model where all C paths are dropped; −2ll= minus twice the log likelihood; df= degrees of freedom; AIC= Akaike’s information criterion; Δ -2ll =difference in -2ll value; Δ df= difference in degrees of freedom; p= p-value; The best fitting models are marked as bold, selected based on the principle of parsimony and lowest AIC and -2ll value. A difference in AIC between two models of 2 or less, provides equivalent support for both models, in which case the most parsimonious model (i.e. with lowest number of parameters) was chosen, a difference of 3 indicates that the lower AIC model has considerably more support, and a difference of more than 10, indicates that the lower AIC model is a substantially better fit compared to the higher AIC model. | | | | | | | | |


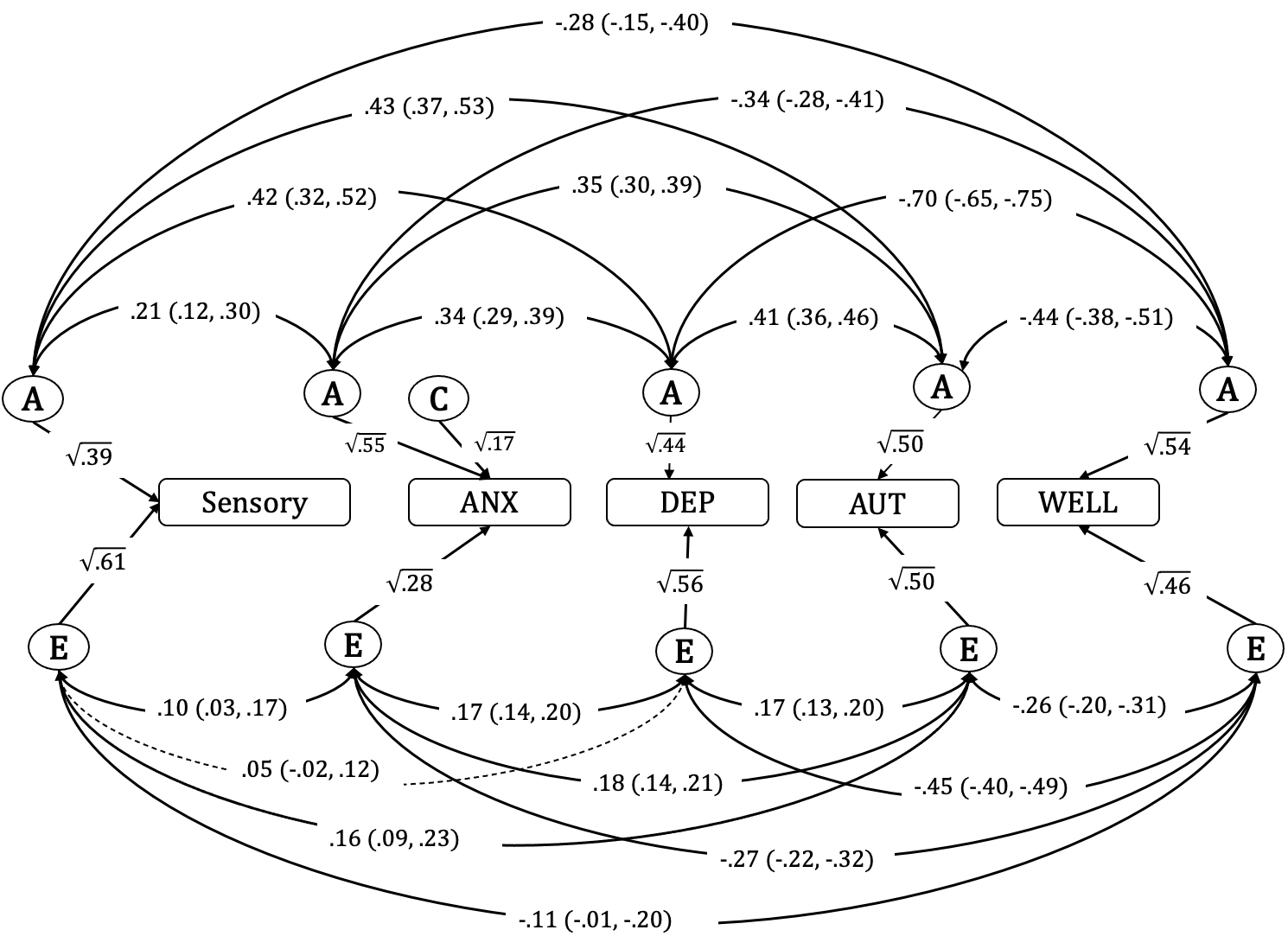


**Figure S4.** Model 1 (correlated factors solution of the Cholesky decomposition model) for **Sensory sensitivity factor.** ANX=anxiety symptoms; AUT=autistic traits; DEP=depressive symptoms; WELL=subjective wellbeing factor; A= additive genetic influences; C= common environmental influences; E= unique environmental influences. 95% Confidence intervals are shown in parentheses. Straight arrows represent contributions from ACE components to each variable, and curved arrows represent correlations between AE components for variables.

| **Table S7b. Model 1 (**correlated factors solution of the Cholesky decomposition model**) estimates for Sensory sensitivity factor** | | | | | | |
| --- | --- | --- | --- | --- | --- | --- |
|  |  | **rPh** | **rPhA** | **rPhE** | **% rPhA** | **% rPhE** |
| **Sensory** | Anx | .14 (.10, .17) | .10 (.06, .14) | .04 (.01, .07) | 71 | 29 |
|  | Dep | .20 (.17, .24) | .17 (.13, .22) | .03 (-.01, .06) | 85 | 15 |
|  | AUT | .28 (.25, .31) | .19 (.15, .23) | .09 (.05, .13) | 68 | 32 |
|  | Well | -.18 (-.23, -.14) | -.13 (-.18, -.12) | -.06 (-.11, -.01) | 72 | 28 |
| **Anxiety symptoms** | Dep | .23 (.21, .25) | .17 (.14, .19) | .07 (.06, .08) | 74 | 26 |
|  | AUT | .25 (.23, .27) | .18 (.16, .20) | .07 (.05, .08) | 72 | 28 |
|  | Well | -.28 (-.31, -.25) | -.19 (-.22, -.15) | -.10 (-.12, -.08) | 68 | 32 |
| **Depressive symptoms** | AUT | .28 (.26, .30) | .19 (.17, .22) | .09 (.07, .11) | 68 | 32 |
|  | Well | -.57 (-.59, -.55) | -.34 (-.37, -.31) | -.23 (-.25, -.20) | 60 | 40 |
| **Autistic traits** | Well | -.35 (-.38, -.33) | -.23 (-.27, -.20) | -.12 (-.15, -.10) | 66 | 34 |
| Results from the best fitting AE model (AE2); Sensory= Sensory sensitivity factor; Anx=anxiety symptoms; Dep=depressive symptoms; AUT= autistic traits; Well= subjective wellbeing; rPh= phenotypic correlation; rPhA= phenotypic correlation due to correlating genetic effects; rPhE= phenotypic correlation due to correlating environmental effects; % rPhA= percentage of phenotypic correlation due to correlating genetic effects; % rPhE= percentage of phenotypic correlation due to correlating environmental effects; Percentages are rounded up | | | | | | |

| **Table S8a**. Twin correlations for **Aesthetic sensitivity factor** and psychological wellbeing | | | | |
| --- | --- | --- | --- | --- |
|  |  | Aesthetic | wellbeing | Aesthetic-wellbeing |
| rMZ |  | .39 (.31, .45) | .47 (.42, .53) |  |
| rDZ |  | .12 (.06, .19) | .26 (.20, .31) |  |
| rPh |  |  |  | .23 (.18, .29) |
| MZ xtxt |  |  |  | .10 (.02, .17) |
| DZ xtxt |  |  |  | .05 (-.02, .12) |
| Aesthetic =Aesthetic sensitivity factor; wellbeing =psychological wellbeing factor score; rMZ= monozygotic (MZ) twins correlations; rDZ=dizygotic (DZ) twins correlation; xtxt=cross-twin cross-trait correlations; rPh= phenotypic correlation | | | | |

**Table S8b**. Bivariate Cholesky AE model estimates for **Aesthetic sensitivity factor**

|  | **A** | **E** | **Ra** | **RE** | **rphA** | **rphE** |
| --- | --- | --- | --- | --- | --- | --- |
| Aesthetic | .35 (.29, .41) | .65 (.59, .71) |  |  |  |  |
| well | .48 (.43, .53) | .52 (.47, .57) |  |  |  |  |
| Aesthetic -well |  |  | .24 (.07, .40) | .23 (.13, .33) | .10 (.03, .17) | .13 (.07, .19) |

Aesthetic=Aesthetic sensitivity factor; wellbeing =psychological wellbeing factor score; A= additive genetic effects; E=unique environmental effects; rPhA= phenotypic correlation due to correlating genetic effects; rPhE= phenotypic correlation due to correlating environmental effects

| **Table S8c**. Cholesky AE model fit results for **Aesthetic sensitivity factor** | | | | | | | | |
| --- | --- | --- | --- | --- | --- | --- | --- | --- |
| **base** | **comparison** | **ep** | **minus2LL** | **df** | **AIC** | **Δ -2ll** | **Δ df** | **p** |
| Sat |  | 28 | 18299.70 | 6643 | 18355.70 |  |  |  |
| Sat | Sub | 11 | 18341.60 | 6660 | 18363.60 | 41.90 | 17 | 7E-04 |
| Sub |  | 11 | 18341.60 | 6660 | 18363.60 |  |  |  |
| Sub | ACE | 11 | 18345.45 | 6660 | 18367.45 | 3.85 | 0 | *NA* |
| Sub | AE | 8 | 18345.89 | 6663 | 18361.89 | 4.29 | 3 | 0.23 |
| ACE |  | 11 | 18345.45 | 6660 | 18367.45 | 3.85 | 0 |  |
| **ACE** | **AE** | **8** | **18345.89** | **6663** | **18361.89** | **0.44** | **3** | **0.93** |
| Sat= Saturated model with maximum number of parameters describing the data; Sub=constrained sub-model of the fully saturated model, testing the assumptions of twin design, with means and variances equated across twins and zygosity; ACE= univariate ACE model; AE= sub-model of the ACE model where all C paths are dropped; −2ll= minus twice the log likelihood; df= degrees of freedom; AIC= Akaike’s information criterion; Δ -2ll =difference in -2ll value; Δ df= difference in degrees of freedom; p= p-value; The best fitting models are marked as bold, selected based on the principle of parsimony and lowest AIC and -2ll value. A difference in AIC between two models of 2 or less, provides equivalent support for both models, in which case the most parsimonious model (i.e. with lowest number of parameters) was chosen, a difference of 3 indicates that the lower AIC model has considerably more support, and a difference of more than 10, indicates that the lower AIC model is a substantially better fit compared to the higher AIC model. | | | | | | | | |


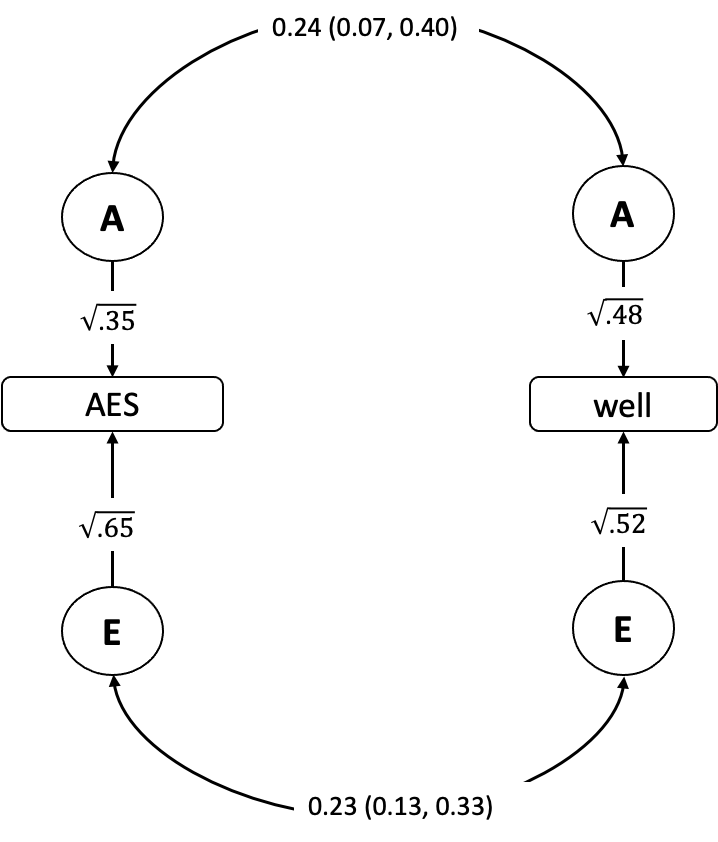


**Figure S5.** Bivariate Cholesky decomposition model for aesthetic sensitivity factor (AES) and subjective wellbeing (well). A= additive genetic influences; E= unique environmental influences.

**Model 2:** ordered-correlated factors solution of the Cholesky decomposition.

| **Table S9**. Model 2 (ordered- correlated factors solution of the Cholesky decomposition model) fit results for sensitivity, anxiety and depressive symptoms, autistic traits and wellbeing | | | | | | | | |
| --- | --- | --- | --- | --- | --- | --- | --- | --- |
| **base** | **comparison** | **ep** | **minus2LL** | **df** | **AIC** | **Δ -2ll** | **Δ df** | **p** |
| Sub |  | 50 | 130107.30 | 36472 | 130207.30 |  |  |  |
| Sub | AE2 | 36 | 130145.10 | 36486 | 130217.10 | 37.78 | 14 | 6E-04 |
| ACE |  | 50 | 130129.00 | 36472 | 130229.00 |  |  |  |
| ACE | AE2 | 36 | 130145.10 | 36486 | 130217.10 | 16.17 | 14 | 0.30 |
| Sat= Saturated model with maximum number of parameters describing the data; Sub=constrained sub-model of the fully saturated model, testing the assumptions of twin design, with means and variances equated across twins and zygosity; ACE= ACE model; AE2= sub-model of the ACE model where all C paths expect for variable 2 are dropped; −2ll= minus twice the log likelihood; df= degrees of freedom; AIC= Akaike’s information criterion; Δ -2ll =difference in -2ll value; Δ df= difference in degrees of freedom; p= p-value; The best fitting models are marked as bold, selected based on the principle of parsimony and lowest AIC and -2ll value. A difference in AIC between two models of 2 or less, provides equivalent support for both models, in which case the most parsimonious model (i.e. with lowest number of parameters) was chosen, a difference of 3 indicates that the lower AIC model has considerably more support, and a difference of more than 10, indicates that the lower AIC model is a substantially better fit compared to the higher AIC model. | | | | | | | | |

**Sensitivity analyses: Model 2**

Two sensitivity analyses were run for Model 2. In the first, the parent-report measure of anxiety in the model was replaced with a self-report measure, the Childhood Anxiety Sensitivity Index (**CASI**; Silverman, Fleisig, Rabian, & Peterson, 1991). This measure mainly captures adolescents’ awareness of, and tendency to, negatively interpret symptoms of anxiety (Chorpita, Albano, & Barlow, 1996). Second, the total environmental sensitivity score was replaced with “Excitation” and “Sensory” factors in the model to examine their association with mental health outcomes and wellbeing.

| **Table S10.**  Model 2 (ordered-correlated factors solution of the Cholesky decomposition model) fit results using **CASI for anxiety symptoms** | | | | | | | | |
| --- | --- | --- | --- | --- | --- | --- | --- | --- |
| **base** | **comparison** | **ep** | **minus2LL** | **df** | **AIC** | **Δ -2ll** | **Δ df** | **p** |
| Sub |  | 50 | 129282.50 | 36427 | 129382.50 |  |  |  |
| Sub | ACE | 50 | 129305.80 | 36427 | 129405.80 | 23.35 | 0 | *NA* |
| Sub | AE2 | 36 | 129322.20 | 36441 | 129394.20 | 39.78 | 14 | 3E-04 |
| Sub | AE | 35 | 129322.30 | 36442 | 129392.30 | 39.86 | 15 | 5E-04 |
| ACE |  | 50 | 129305.80 | 36427 | 129405.80 |  |  |  |
| ACE | AE2 | 36 | 129322.20 | 36441 | 129394.20 | 16.42 | 14 | 0.29 |
| ACE | AE | 35 | 129322.30 | 36442 | 129392.30 | 16.50 | 15 | 0.35 |
| AE2 | AE | 35 | 129322.30 | 36442 | 129392.30 | 0.08 | 1 | 0.78 |
| Sat= Saturated model with maximum number of parameters describing the data; Sub=constrained sub-model of the fully saturated model, testing the assumptions of twin design, with means and variances equated across twins and zygosity; ACE= ACE model; AE2= sub-model of the ACE model where all C paths expect for variable 2 are dropped; AE= sub-model of the AE model where all C paths are dropped; ; −2ll= minus twice the log likelihood; df= degrees of freedom; AIC= Akaike’s information criterion; Δ -2ll =difference in -2ll value; Δ df= difference in degrees of freedom; p= p-value; The best fitting models are marked as bold, selected based on the principle of parsimony and lowest AIC and -2ll value. A difference in AIC between two models of 2 or less, provides equivalent support for both models, in which case the most parsimonious model (i.e. with lowest number of parameters) was chosen, a difference of 3 indicates that the lower AIC model has considerably more support, and a difference of more than 10, indicates that the lower AIC model is a substantially better fit compared to the higher AIC model. | | | | | | | | |


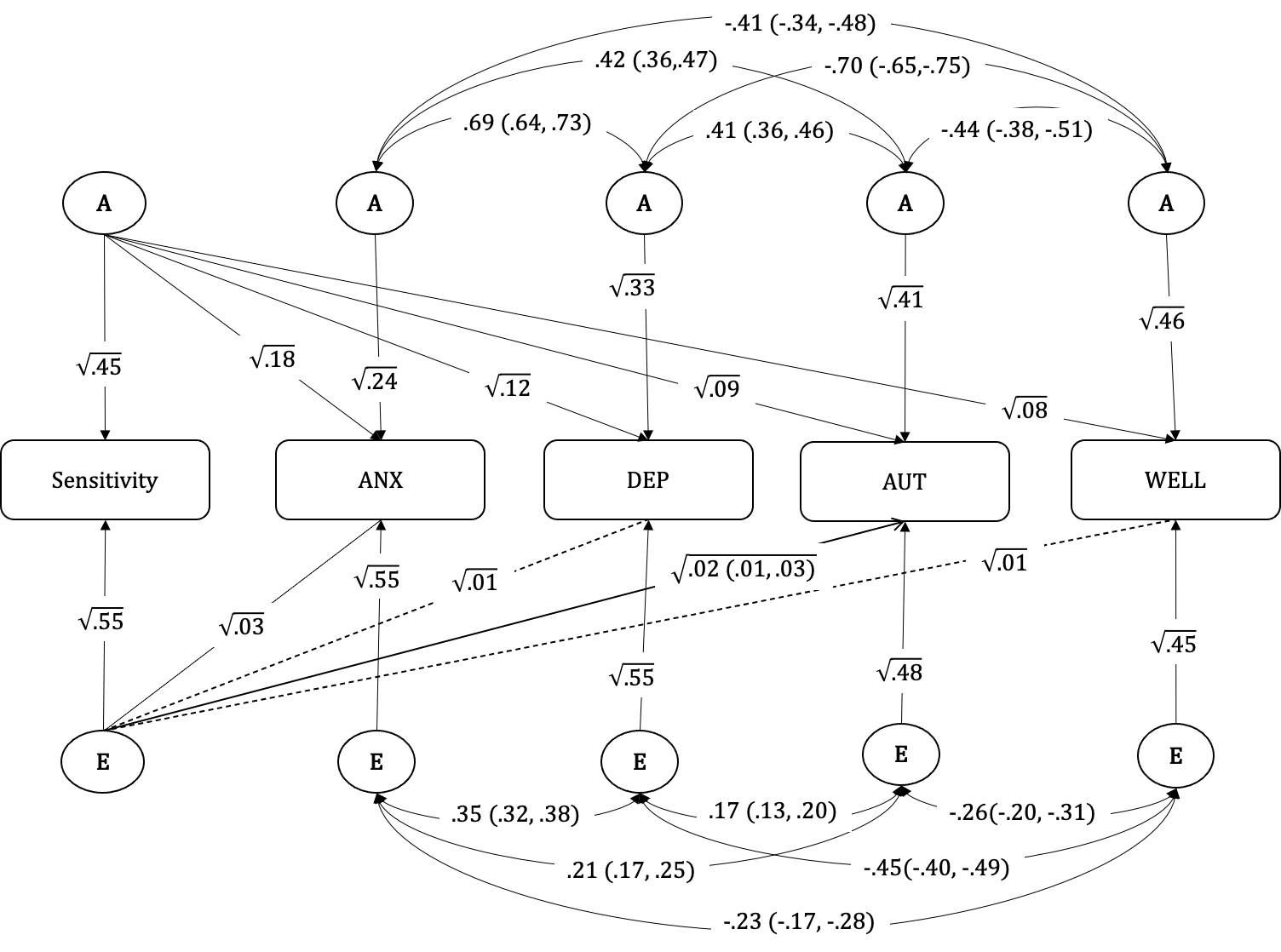


**Figure S6.** Model 2 (ordered-correlated factors solution of the Cholesky decomposition model) using **CASI for anxiety symptoms**. Sensitivity=environmental sensitivity; ANX=anxiety symptoms; AUT=autistic traits; DEP=depressive symptoms; WELL=subjective wellbeing score; The dotted lines indicate non-significant path estimates. A= additive genetic influences; C= common environmental influences; E= unique environmental influences. 95% Confidence intervals are shown in parentheses. Straight arrows represent contributions from ACE components to each variable, and curved arrows represent correlations between AE components for variables.

| **Table S11**. Model 2 (ordered-correlated factors solution of the Cholesky decomposition model) fit results for **sensory sensitivity factor** | | | | | | | | |
| --- | --- | --- | --- | --- | --- | --- | --- | --- |
| **base** | **comparison** | **ep** | **minus2LL** | **df** | **AIC** | **Δ -2ll** | **Δ df** | **p** |
| Sub |  | 50 | 132429.00 | 36472 | 132529.00 |  |  |  |
| Sub | AE2 | 36 | 132469.60 | 36486 | 132541.60 | 40.61 | 14 | 2E-04 |
| ACE |  | 50 | 132452.60 | 36472 | 132552.60 |  |  |  |
| ACE | AE2 | 36 | 132469.60 | 36486 | 132541.60 | 17.00 | 14 | 0.26 |
| Sat= Saturated model with maximum number of parameters describing the data; Sub=constrained sub-model of the fully saturated model, testing the assumptions of twin design, with means and variances equated across twins and zygosity; ACE= ACE model; AE2= sub-model of the ACE model where all C paths expect for variable 2 are dropped; −2ll= minus twice the log likelihood; df= degrees of freedom; AIC= Akaike’s information criterion; Δ -2ll =difference in -2ll value; Δ df= difference in degrees of freedom; p= p-value; The best fitting models are marked as bold, selected based on the principle of parsimony and lowest AIC and -2ll value. A difference in AIC between two models of 2 or less, provides equivalent support for both models, in which case the most parsimonious model (i.e. with lowest number of parameters) was chosen, a difference of 3 indicates that the lower AIC model has considerably more support, and a difference of more than 10, indicates that the lower AIC model is a substantially better fit compared to the higher AIC model. | | | | | | | | |


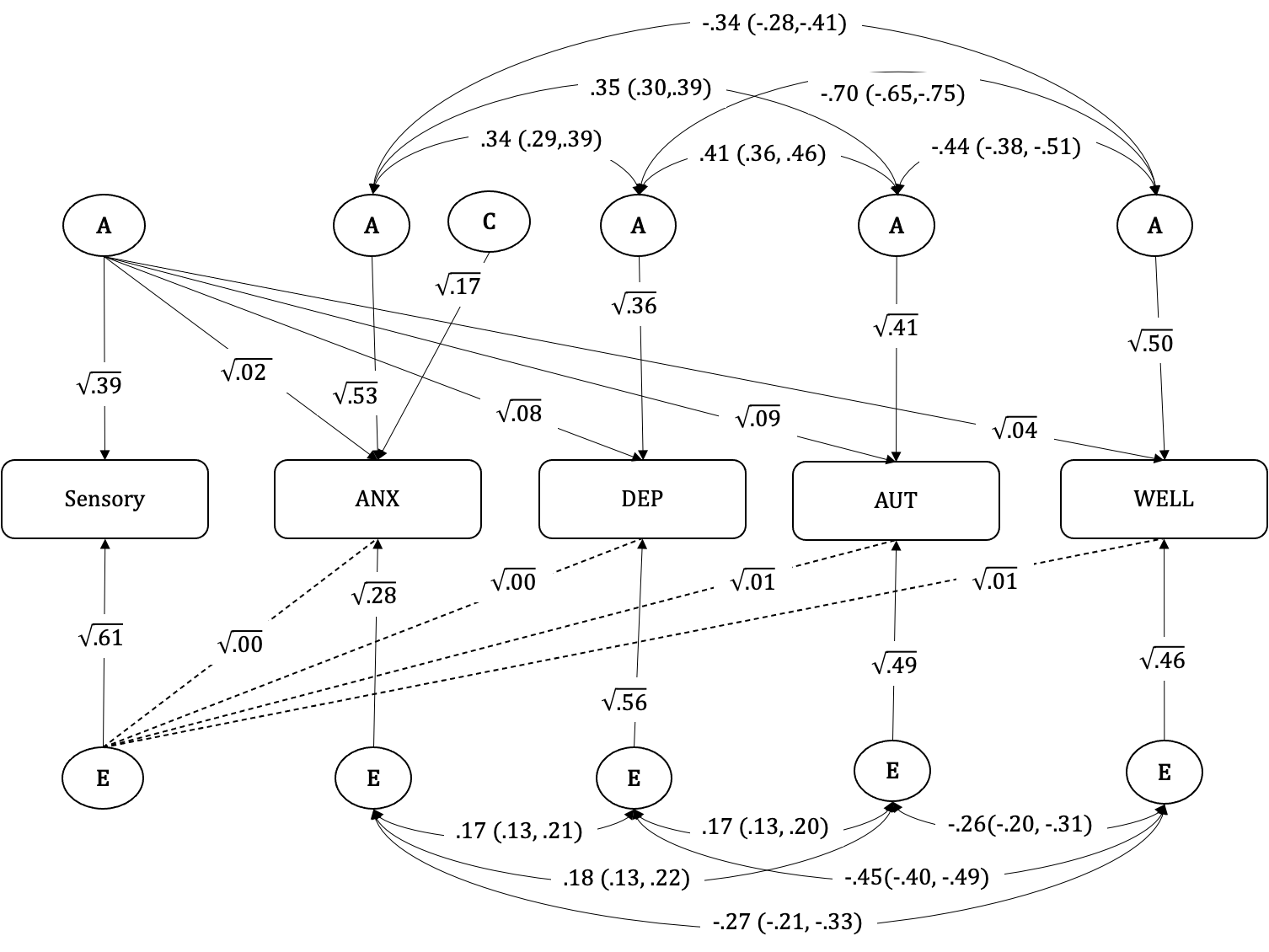


**Figure S7.** Model 2 (ordered-correlated factors solution of the Cholesky decomposition model) for **sensory sensitivity factor.** Sensitivity=environmental sensitivity; ANX=anxiety symptoms; AUT=autistic traits; DEP=depressive symptoms; WELL=subjective wellbeing score; The dotted lines indicate non-significant path estimates. A= additive genetic influences; C= common environmental influences; E= unique environmental influences. 95% Confidence intervals are shown in parentheses. Straight arrows represent contributions from ACE components to each variable, and curved arrows represent correlations between AE components for variables.


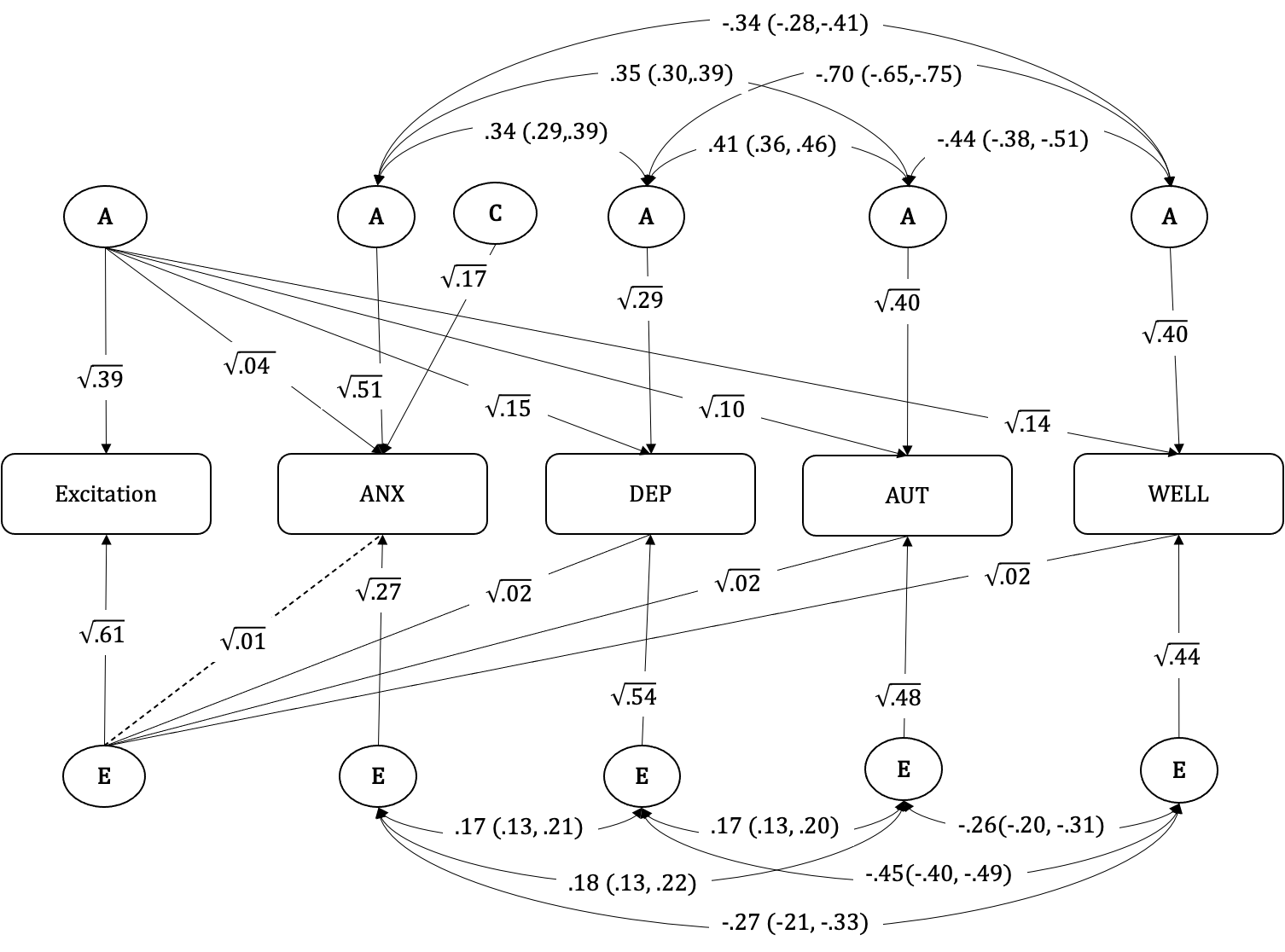


**Figure S8.** Model 2 (ordered-correlated factors solution of the Cholesky decomposition model) for **Excitation sensitivity factor.** Sensitivity=environmental sensitivity; ANX=anxiety symptoms; AUT=autistic traits; DEP=depressive symptoms; WELL=subjective wellbeing score; The dotted lines indicate non-significant path estimates. A= additive genetic influences; C= common environmental influences; E= unique environmental influences. 95% Confidence intervals are shown in parentheses. Straight arrows represent contributions from ACE components to each variable, and curved arrows represent correlations between AE components for variables.

REFERENCES

Duckworth, A. L., Peterson, C., Matthews, M. D., & Kelly, D. R. (2007). Grit: perseverance and passion for long-term goals. *J Pers Soc Psychol, 92*(6), 1087-1101. doi:10.1037/0022-3514.92.6.1087

Duckworth, A. L., & Quinn, P. D. (2009). Development and validation of the short grit scale (grit-s). *J Pers Assess, 91*(2), 166-174. doi:10.1080/00223890802634290

Kashdan, T. B., Rose, P., & Fincham, F. D. (2004). Curiosity and exploration: facilitating positive subjective experiences and personal growth opportunities. *J Pers Assess, 82*(3), 291-305. doi:10.1207/s15327752jpa8203_05

Lyubomirsky, S., & Lepper, H. S. (1999). A measure of subjective happiness: Preliminary reliability and construct validation. *Social Indicators Research, 46*(2), 137-155. doi:10.1023/a:1006824100041

McCullough, M. E., Emmons, R. A., & Tsang, J. A. (2002). The grateful disposition: a conceptual and empirical topography. *J Pers Soc Psychol, 82*(1), 112-127. doi:10.1037//0022-3514.82.1.112

Peel, A. J., Oginni, O., Assary, E., Krebs, G., Lockhart, C., McGregor, T., . . . Eley, T. C. (2023). A multivariate genetic analysis of anxiety sensitivity, environmental sensitivity and reported life events in adolescents. *J Child Psychol Psychiatry, 64*(2), 289-298. doi:10.1111/jcpp.13725

Scheier, M. F., Carver, C. S., & Bridges, M. W. (1994). Distinguishing Optimism from Neuroticism (and Trait Anxiety, Self-mastery, And Self-esteem) - A Reevaluation of the Life Orientation Test. *Journal of Personality and Social Psychology, 67*(6), 1063-1078. doi:10.1037/0022-3514.67.6.1063

Seligson, J. L., Huebner, E. S., & Valois, R. F. (2003). Preliminary validation of the Brief Multidimensional Students' Life Satisfaction Scale (BMSLSS). *Social Indicators Research, 61*, 121-145.

Snyder, C. R., Hoza, B., Pelham, W. E., Rapoff, M., Ware, L., Danovsky, M., . . . Stahl, K. J. (1997). The development and validation of the Children's Hope Scale. *J Pediatr Psychol, 22*(3), 399-421. doi:10.1093/jpepsy/22.3.399
